# Mechanical Stimuli‐Induced Manipulation of Malignant Behavior in Bioprinted Cancer Microtissues via PI3K/NF‐κB Activation

**DOI:** 10.1002/advs.202518295

**Published:** 2026-01-04

**Authors:** Seok‐Hyeon Lee, Jeongho Lee, Min‐Seo Choi, Minjun Ahn, Sik Yoon, Dongjun Lee, Sae‐Ock Oh, Won‐Woo Cho, Byoung Soo Kim

**Affiliations:** ^1^ School of Biomedical Convergence Engineering Pusan National University Yangsan Republic of Korea; ^2^ Medical Research Institute Pusan National University Yangsan Republic of Korea; ^3^ Department of Anatomy School of Medicine Pusan National University Yangsan Republic of Korea; ^4^ Department of Convergence Medicine School of Medicine Pusan National University Yangsan Republic of Korea; ^5^ Transplantation Research Center Research Institute for Convergence of Biomedical Science and Technology Pusan National University Yangsan Hospital Yangsan Republic of Korea; ^6^ Department of Biomedical Engineering Yonsei University Wonju Republic of Korea; ^7^ Research Institute for Convergence of Biomedical Science and Technology Pusan National University Yangsan Hospital Yangsan Republic of Korea

**Keywords:** in‐bath 3D bioprinting, matrix stiffness, prostate cancer

## Abstract

Increased matrix stiffness within tumor microenvironments (TMEs) significantly influences cancer progression and gene expression, contributing to drug resistance and poor clinical outcomes. Studies demonstrate a strong correlation between nuclear factor kappa B (NF‐κB) upregulation and prostate cancer malignancy. However, the mechanisms by which the mechanical stress within the TME activates NF‐κB remain underexplored. This study developed a prostate cancer spheroid model using an in‐bath 3D bioprinting technique. Cancer spheroids were printed within a bespoke hydrogel bath with tunable stiffness, facilitating the investigation of the relationship between mechanical cues and oncogenic behavior. Increased hydrogel stiffness promoted spheroid compaction, induction of epithelial–mesenchymal transition (EMT) and stemness programs, and elevated drug resistance. Transcriptomic analysis revealed that the phosphoinositide 3‐kinase (PI3K) pathway is most enriched under mechanical stress. Findings demonstrated that increased extracellular matrix stiffness activated PI3K/NF‐κB signaling through mechanotransduction. Pharmacological inhibition of PI3K suppressed NF‐κB nuclear translocation and enhanced chemotherapy efficacy. The bespoke hydrogel effectively recapitulated the mechanical environment of prostate cancer, indicating the pivotal role of PI3K/NF‐κB signaling in regulating prostate cancer malignancy under mechanical stimulation. This suggests a promising therapeutic avenue for improving treatment outcomes.

## Graphical abstract

A mechanically tunable ECM platform is created by integrating dECM–alginate matrices with in‐bath tumor printing. The mechanically tunable ECM environment drives tumors to acquire hypoxia, mechanotransduction signaling, metastatic traits, and drug‐resistant phenotypes that collectively promote malignant transformation. Targeted screening within this platform identifies specific therapeutic responses, demonstrating the platform's utility for evaluating stiffness‐dependent malignancy and drug screening.

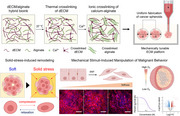

## 1 Introduction

Prostate cancer lesions exhibit significantly higher tissue stiffness than benign counterparts, correlating with elevated Gleason scores and aggressiveness [1, 2, 3]. This increased matrix stiffness alters gene and protein expression, enhancing chemoresistance, and correlates strongly with aggressive clinical presentation. Among the key mechanotransduction pathways, nuclear factor kappa B (NF‐κB) has emerged as a central mediator, linking intrinsic oncogenic signaling to mechanical stress [4, 5]. When stimulated, NF‐κB undergoes nuclear translocation and drives gene transcription, promoting proliferation, angiogenesis, and epithelial–mesenchymal transition (EMT) in prostate cancer [6, 7]. However, the precise mechanisms by which mechanical stress within the tumor microenvironment (TME) activates

NF‐κB remain elusive. This limits the efforts to develop stiffness‐targeted precision therapies. To model mechanotransduction in vitro, hydrogel‐based 3D systems were developed to recapitulate solid TMEs [7, 8, 9, 10]. Cross‐linked hydrogel matrices mimic certain aspects of the extracellular matrix (ECM), facilitating studies of cell–matrix interactions. Yet, conventional hydrogels lack native ECM complexity, exhibit mechanical tunability, and suffer from poor scalability and reproducibility. These limitations hinder their applicability as robust platforms for empirical oncogenic research and precise prediction of drug responses.

This study developed a customized hydrogel composed of decellularized extracellular matrix (dECM) and alginate, enabling precise modulation of stiffness from 5 to 55 kPa while retaining native biochemical cues. An in‐bath 3D bioprinting strategy facilitated rapid and reproducible production of prostate cancer spheroids within these hydrogels, faithfully replicating the mechanical conditions of the TME. Increased stiffness increased malignant traits, including activation of integrin signaling, nuclear Yes‐Associated Protein (YAP) translocation, EMT, and chemoresistance. Transcriptomic analysis via next‐generation sequencing revealed that stiff environment genes are linked to tumor progression, as validated by Gene Ontology (GO) analysis and Gene Set Enrichment Analysis (GSEA). Kyoto Encyclopedia of Genes and Genomes (KEGG) pathway analysis identified phosphoinositide 3‐kinase (PI3K) signaling closely linked to NF‐κB as the most prominently activated under mechanical stress. Pharmacological inhibition of PI3K suppressed NF‐κB nuclear translocation and reversed stiffness‐induced malignancy even under stiff conditions. This tunable cancer model enables systematic investigation of the underlying mechanisms of stiffness‐induced therapeutic resistance and serves as a reliable drug screening platform for evaluating therapeutic strategies against aggressive cancers.

## 2 Results and Discussion

### 2.1 Development of Stiffness‐Tunable Hybrid Bioink

**FIGURE 1:**
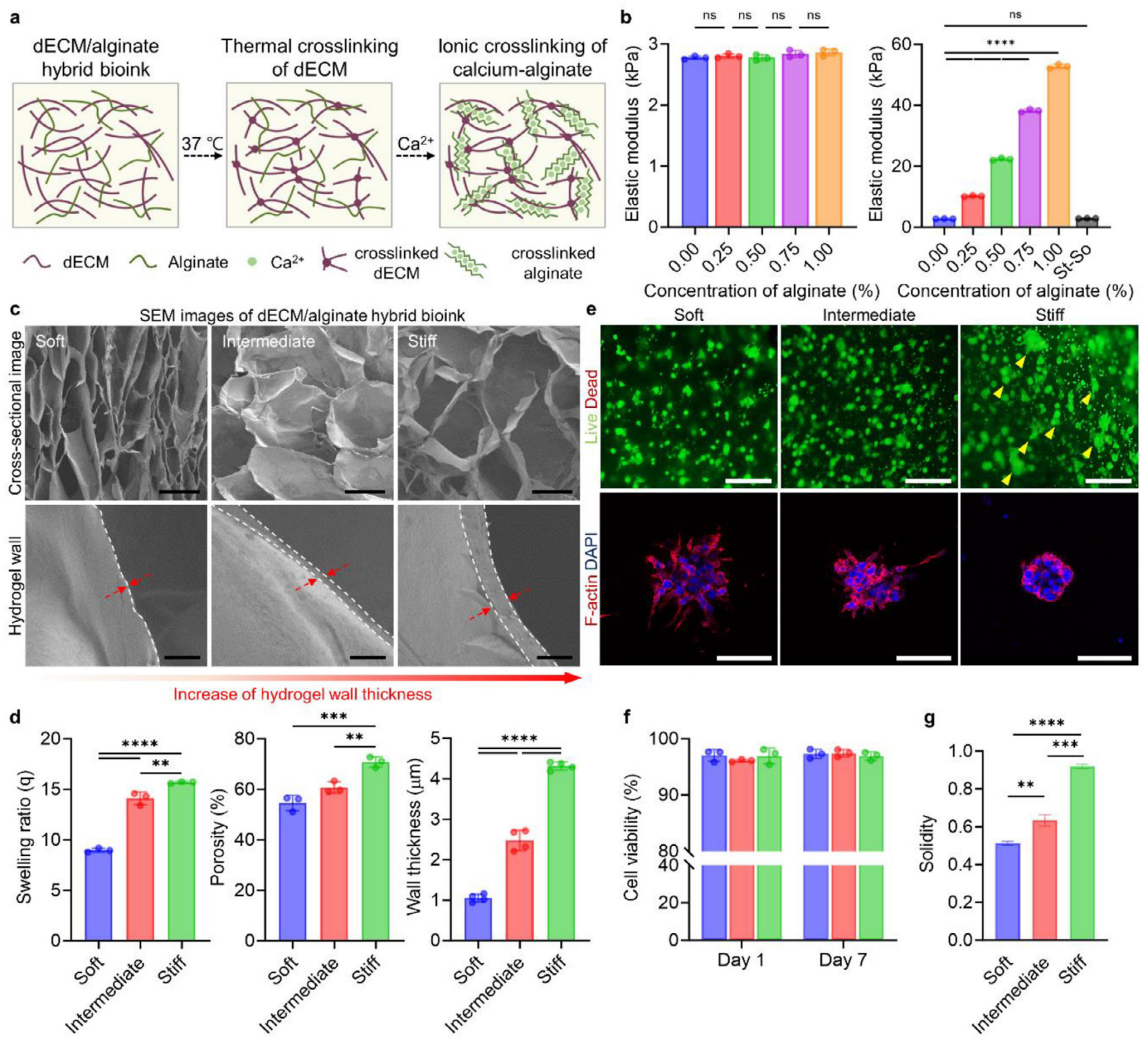
Characterization of stiffness‐tunable hybrid bioink. (a) Schematic of a hybrid bioink composed of dECM and alginate, representing structural characteristics of each component. (b) Quantification of elastic modulus of soft, intermediate, and stiff hydrogel groups after thermal cross‐linking (left) and after subsequent ionic cross‐linking (right) (*n* = 3). (c) Cross‐sectional SEM images of soft, intermediate, and stiff hydrogel groups. Upper panels show overall structure, and lower panels present magnified images of hydrogel walls. Scale bars: 250 µm (upper) and 10 µm (lower). (d) Quantification of swelling ratio, porosity, and pore wall thickness for soft, intermediate, and stiff hydrogel groups (*n* = 3). (e) Fluorescence microscopy images after 7 days of culture. Upper panels show live/dead staining (live: green, dead: red). Lower panels show F‐actin (red) and DAPI (blue) staining to visualize the cytoskeletal organization and morphology of the cells within clusters. Scale bars: 150 µm (upper) and 100 µm (lower) (*n* = 3). (f) Quantification of cell viability in soft, intermediate, and stiff hydrogel groups on days 1 and 7 postencapsulation (*n* = 3). (g) Quantification of solidity of cell clusters formed in soft, intermediate, and stiff hydrogel after 7 days (*n* = 3). The error bars represent mean ± SD. Statistical significance was assessed using one‐way ANOVA (*p < 0.05, **p < 0.01, ***p < 0.001, ****p < 0.0001).

To mimic the solid TME that drives malignant progression, a hybrid bioink incorporating ECM components with tunable stiffness was developed (Figure 1a). The hybrid bioink utilized dECM as the base material, providing key ECM components such as collagen and glycosaminoglycans (GAGs) and minimizing immune reactions by retaining DNA levels below the widely accepted threshold of 50 ng/mg dry ECM (Figure S1a) [8, 9, 10, 11, 12]. Stiffness was modulated by adding sodium alginate, a natural polysaccharide widely used in hydrogels [13, 14, 15]. To maintain a uniform biochemical composition, the dECM concentration was fixed at 1.0% and stiffness was modulated by varying the alginate concentration. Following a two‐step cross‐linking process thermal and ion all hybrid bioink formulations exhibited a uniform elastic modulus after thermal cross‐linking. However, after the secondary ion cross‐linking, stiffness increases proportionally with alginate concentration (Figure 1b).

Given the heterogeneity in ECM stiffness in prostate cancer patients [16, 17, 18], the study aimed to simulate physiologically relevant stiffness gradients to investigate stiffness‐associated alterations [19]. The formulations are categorized into three groups: soft (1.0% dECM), corresponding to the stiffness of benign or normal prostate tissue typically reported in the 2–10 kPa range [2.8 ± 0.12 kPa], intermediate (1.0% dECM + 0.5% alginate), representing a transitional stiffness level that reflects stromal remodeling and early desmoplastic changes [22.7 ± 0.23 kPa] and stiff (1.0% dECM + 1.0% alginate), reflecting clinically reported thresholds of malignant prostate tissue stiffness, typically ≥35 kPa [53.5 ± 0.33 kPa] [20, 21, 22]. To evaluate whether the epigenetic changes induced by stiffness were directly attributable to the matrix mechanics and reversible, an additional group was introduced, designated as stiff → soft (St → So) (Figure S2). In this group, bioink was treated with alginate lyase on day 3 of culture to digest the alginate components, reducing stiffness of the stiff group [53.5 ± 0.33 kPa] to match the soft group [2.8 ± 0.12 kPa].

Subsequent examination using scanning electron microscopy (SEM) analysis characterized the porous architecture of all the hybrid bioinks groups (Figure 1c). Porosity increased with higher alginate concentration, despite expectations of reduced porosity. This behavior is attributed to contraction and reorganization during ion cross‐linking, which varies with material properties [23, 24, 25]. Furthermore, higher alginate concentration thickened pore walls, resulting in a more robust and stable structure (Figure 1d). Swelling ratio analysis corroborated the observed increase in porosity. These findings confirm that the developed hybrid bioink offers tunable stiffness, defined microstructural characteristics, and a porous architecture conducive for cell culture.

The cytocompatibility of the hybrid bioink and its ability to influence cancer cell behavior were evaluated by encapsulating single cancer cells within each formulation under identical seeding densities. The cells used in this study were the prostate cancer cell line PC3, which is widely established in mechanobiology research and shows robust stiffness‐dependent responses, making it a suitable model for investigating mechanotransduction‐driven malignancy [26, 27]. Cells were cultured for 7 days to monitor proliferation (Figure 1e). Live–dead staining revealed minimal cell death across all formulations (Figure 1f). Cell morphology and organization varied with stiffness. Stiffer matrices promoted the formation of compact cellular clusters (Figure 1g), likely due to solid stress, which can induce hypoxia within the TME. These findings indicate that the tunable stiffness, porous architecture, and cytocompatibility of the hybrid bioink provide a reliable platform for investigating stiffness‐mediated cellular behaviors.

### 2.2 Mechanical Remodeling of Tumor Spheroids in a Stiffness‐Tunable System

**FIGURE 2:**
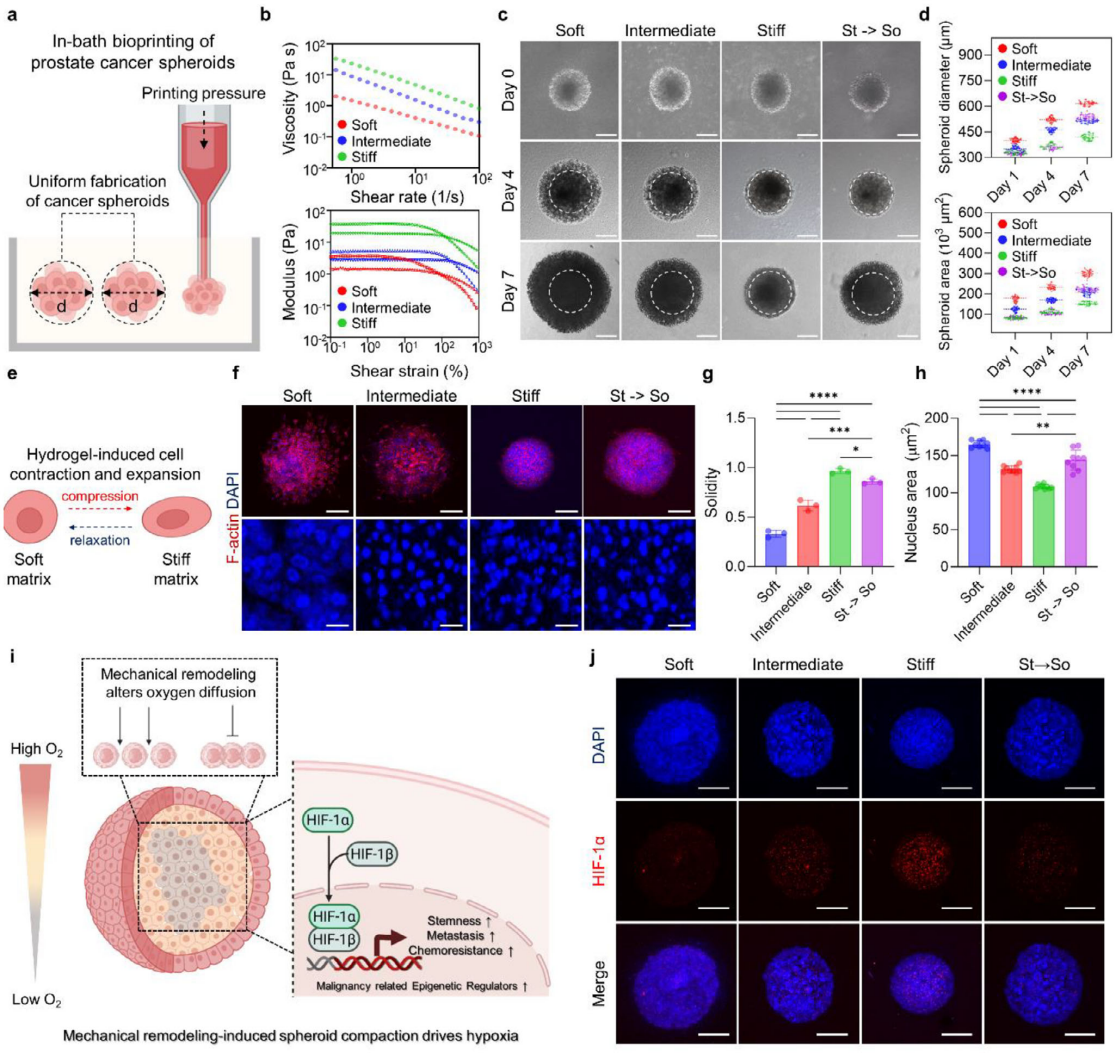
Mechanical remodeling and compaction of tumor spheroids under tunable stiffness conditions. (a) Schematic showing accuracy of in‐bath bioprinting and spheroid size modulation by controlling printing pressure. (b) Rheological measurements demonstrating shear‐thinning behavior (upper) and Bingham plastic characteristics (lower) of hybrid bioink. (c) Optical microscopy images of spheroids formed in soft, intermediate, stiff, and St → So groups at days 0, 4, and 7. (d) Quantification of spheroid diameter (upper) and area (lower) across soft, intermediate, stiff, and St → So groups (*n* = 80). (e) Schematic illustrating hydrogel‐induced cell contraction and expansion. (f) Confocal images of spheroids at day 7 postprinting. Upper panels show the overall spheroid structure, and lower panels depict DAPI‐stained nuclei in the central region. Scale bars, 150 µm (upper) and 50 µm (lower). (g) Quantification of spheroid solidity (*n* = 3). (h) Quantification of nuclear area (*n* = 10). (i) Schematic showing the formation of a hypoxic environment driven by cell compaction. (j) Representative confocal images of HIF‐1α (red) and DAPI (blue) staining in each group. Scale bars, 200 µm. The error bars represent mean ± SD. Statistical significance was assessed using one‐way ANOVA (*p < 0.05, **p < 0.01, ***p < 0.001, ****p < 0.0001).

**FIGURE 3:**
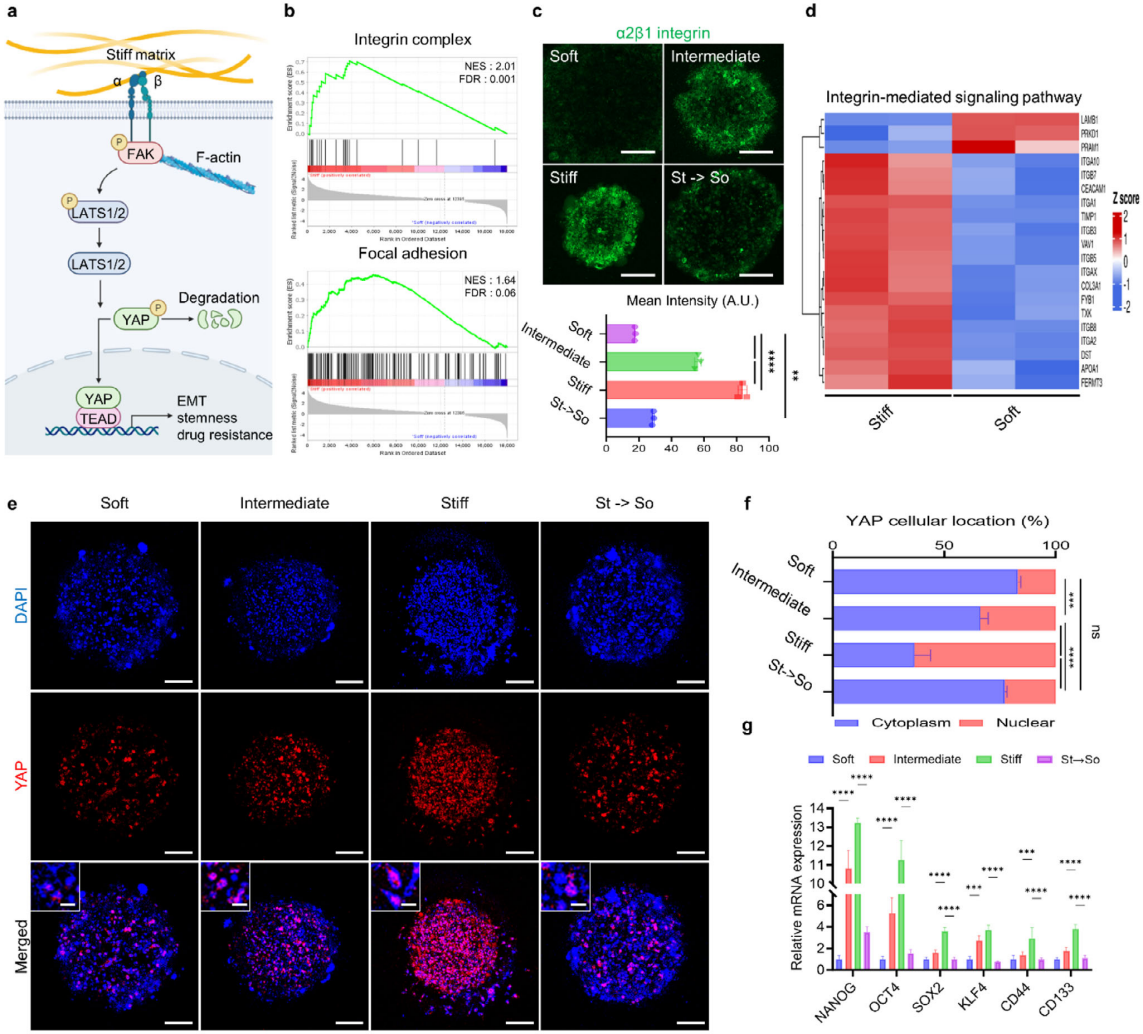
Mechanotransduction pathway profiling in stiffness‐modulated 3D systems. (a) Schematic of the overall process showing nuclear translocation of the representative mechanotransducer YAP. (b) GSEA results showing significant enrichment of the integrin complex (upper; NES = 2.01, FDR = 0.001) and focal adhesion (lower; KEGG pathway: hsa04510, NES = 1.64, FDR = 0.02). (c) Confocal images of α2β1 integrin immunofluorescence staining in soft, intermediate, stiff, and St → So groups (upper), and quantification of mean fluorescence intensity in arbitrary units (A.U.) (lower). Scale bars, 200 µm. (d) Heatmap analysis of integrin‐mediated signaling pathway genes in soft and stiff groups. (e) Confocal images of YAP and DAPI staining in soft, intermediate, stiff, and St → So groups. Scale bars, 100 µm (full view) and 30 µm (enlarged nuclear regions). (f) Quantification of YAP nuclear translocation in soft, intermediate, stiff, and St → So groups (*n* = 3). (g) qPCR results of stemness‐associated markers in soft, intermediate, stiff, and St → So groups (*n* = 3). The error bars represent mean ± SD. Statistical significance was assessed using one‐way ANOVA (*p < 0.05, **p < 0.01, ***p < 0.001, ****p < 0.0001).

In‐bath bioprinting enables direct printing and culture of cells as 3D spheroids within a pregel bioink matrix [8, 10] (Figure 2a and Video S1–S3). To evaluate the suitability of the developed hybrid bioink for this technique, we assessed its rheological properties including shear thinning, Bingham plastic behavior, and shear recovery across the three stiffness groups. All formulations exhibited consistent shear thinning within the defined shear rate range (Figure 2b). This minimizes mechanical resistance during cell extrusion through a needle‐shaped nozzle while ensuring positional stability within the hydrogel. During printing, the support bath exhibits Bingham plastic behavior: it behaves like a solid under low shear stresses, but flows as a viscous fluid at high shear stresses [28]. This dual behavior helps maintain the 3D configuration of deposited cells. Additionally, rapid shear recovery across all bioinks further supports structural fidelity during printing (Figures 2b and 3). Printability was verified through rheological analysis, after which spheroid size was quantified according to printing pressure and established a reference library for consistent size‐controlled spheroid production (Figure S4).

To investigate matrix stiffness‐induced effects, spheroids of the same size for each ink group were printed, using the established size library, and monitored over 7 days. Although all groups exhibited increased spheroid diameter and area (Figure 2c,d and Figure S5), expansion was significantly lower in the stiff group, indicating that high matrix stiffness restricts spatial expansion. Consistent results were also obtained in SNU‐449 and MDA‐MB‐231 cell lines, which were included to evaluate the expandability of the model and to minimize the risk of over‐generalization (Figure S6a). Notably, in the St → So group, this restricted expansion was partially restored, suggesting that the effect was both stiffness‐dependent and reversible. We hypothesized that increased matrix stiffness imposes solid stress on spheroids, limiting expansion and promoting structural solidity (Figure 2e). To validate this hypothesis, the morphological characteristics of spheroids were compared across groups (Figure 2f). Increased matrix stiffness increased spheroid solidity and nuclear compaction (Figure 2g,h). SNU‐449 and MDA‐MB‐231 spheroids exhibited increased solidity under stiff conditions, indicating that stiffness‐induced structural compaction was a shared morphological response (Figure S6b). Interestingly, in the St → So group, these stiffness‐induced effects were partially reversed.

This reversal further supported the hypothesis that the observed morphological changes were primarily driven by the stiffness of the surrounding ECM [29, 30, 31]. Further investigations into stiffness‐induced morphological changes and their impact on cell organization showed enrichment in ECM (GO:0031012), membrane (GO:0016020), and cytoplasm (GO:0005737), suggesting that cells underwent structural modifications in response to a stiff microenvironment. This enrichment reflected broad structural and functional adaptations across cellular compartments in response to mechanical challenges (Figure S7a) [32, 33, 34, 35, 36, 37]. The response to mechanical stimulus heat map further supported the structural changes observed in the GO analysis. Regions with elevated gene expression highlighted key pathways activated under mechanical stress (Figure S7b). These results demonstrated that the stiff microenvironment triggered a coordinated response involving extracellular and intracellular structural components. We hypothesized that such morphological changes induced by the stiff microenvironment, such as spheroid compaction, could enhance oxygen gradients within the spheroid. Hypoxia, a well‐known activator of malignancy‐associated pathways within the TME, is closely linked to such gradients and plays a pivotal role in driving epigenetic and phenotypic changes in cancer cells (Figure 2i). [29, 30, 31].

To assess the impact of matrix stiffness on hypoxia, the expression of hypoxia‐inducible factor 1‐alpha (HIF‐1α) at both the gene and protein levels was examined. HIF‐1α transcript levels were significantly elevated in the stiff group and partially reduced in the St → So condition, where the matrix was softened after initial stiff exposure (Figure S8a). Consistently, immunofluorescence staining showed increased HIF‐1α intensity under stiff conditions, with a visible decrease in the St → So group (Figure 2j). These results further support that solid stress‐induced spheroid compaction directly contributes to the hypoxic response. To determine whether this hypoxic response extended beyond HIF‐1α, the expression patterns of additional hypoxia‐related genes was analyzed. Heatmap analysis revealed coordinated upregulation of multiple hypoxia‐associated targets under stiff conditions, supporting the activation of a broader hypoxia‐driven transcriptional program (Figure S8b). Taken together, using the hybrid bioink based in‐bath bioprinting system, a tunable stiffness 3D culture system capable of applying solid stress to cancer cells was developed, under which increased matrix stiffness‐induced structural deformation, including spheroid compaction and nuclear compression, activated mechanotransduction pathways, and led to a hypoxic response, as indicated by elevated HIF‐1α expression.

## 3 Verification of Mechanotransduction Induced by ECM Stiffness

To investigate the effects of ECM stiffness on mechanotransduction and malignancy, the nuclear translocation of YAP was assessed. Under stiff conditions, integrin clustering activates focal adhesion kinase, inhibiting LATS1/2‐mediated degradation of YAP. Stabilized YAP translocates to the nucleus and interacts with transcription factors such as TEAD, initiating transcriptional programs linked to EMT, stemness, and drug resistance (Figure 3a) [5, 38, 39, 40, 41]. To validate the activation of YAP under stiff conditions, GSEA was conducted to identify pathways enriched in stiffness‐modulated environments. The analysis revealed significant enrichment of pathways directly associated with YAP activity, including integrin complex (NES = 2.01, FDR = 0.001), ECM‐receptor interaction (NES = 1.72, FDR = 0.01), focal adhesion (KEGG pathway: hsa04510; NES = 1.64, FDR = 0.02), and regulation of the actin cytoskeleton (KEGG pathway: hsa04810; NES = 1.52, FDR = 0.06) (Figure 3b and Figure S9a). To confirm further, the molecular functions associated with stiffness‐induced signaling, GO analysis of molecular function was conducted. This analysis revealed significant enrichment in categories such as cytoskeletal protein binding (GO:0008092), tubulin binding (GO:0008017), microtubule binding (GO:0008017), signaling receptor binding (GO:0005102), integrin binding (GO:0005178), calcium ion binding (GO:0005509), receptor ligand activity (GO:0048018), and cytokine activity (GO:0005125). The enrichment of cytoskeletal protein binding, tubulin binding, and microtubule binding suggested that ECM stiffness‐induced cytoskeletal remodeling, which may contribute to changes in cellular morphology and motility. Additionally, the enrichment of integrin binding and signaling receptor binding indicated the activation of mechanosensors, which mediate the conversion of mechanical stimuli into intracellular signaling cascades (Figure S9b) [42, 43, 44]. Given the role of integrins as upstream mediators of YAP signaling, the expression of α2β1 integrins under different stiffness conditions was investigated. Immunofluorescence analysis revealed significantly increased integrin α2β1 expression in the stiff group compared with the soft group, consistent with its established role in sensing mechanical cues and initiating intracellular signaling. Integrin expression decreased in the St → So group, demonstrating that this activation was stiffness‐dependent and reversible (Figure 3c). To further explore stiffness‐induced integrin signaling, heatmap analysis focusing on genes associated with the integrin‐mediated signaling pathway was performed. The stiff group exhibited significant upregulation of multiple integrin‐related signaling components compared with the soft group, indicating that ECM stiffness enhanced integrin‐driven intracellular signaling cascades (Figure 3d).

Notably, the hybrid bioink used in this study, based on dECM, provided an abundant source of collagen, glycosaminoglycans, and other ECM components that served as natural ligands for integrin receptors. This biochemical context, in combination with tunable mechanics, can amplify mechanotransduction within the printed microenvironment (Figure S1a) [45, 46, 47, 48].

Immunofluorescence staining indicated that YAP localization was stiffness‐dependent. In the soft group, YAP was predominantly localized within the cytoplasm, reflecting an inactive state. In contrast, the stiff group exhibited a marked increase in nuclear YAP localization, indicating its activation under stiff conditions. Quantifying the nuclear‐to‐cytoplasmic ratio further confirmed this observation, showing a marked increase in the stiff group compared with the soft group. Notably, in the St → So group, YAP translocated to the cytoplasm following stiffness reduction, demonstrating that this mechanotransduction process was both stiffness‐mediated and reversible (Figure 3e,f).

YAP is a key effector in the Hippo signaling pathway and is linked to regulating cancer stemness. YAP activation under stiff conditions influences stemness‐related transcriptional programs. This was investigated by analyzing the expression of markers, including CD44, NANOG, and OCT4. The stiff group exhibited significantly higher expression of these markers, indicating that YAP nuclear activation under mechanical stress promoted a transcriptional program associated with cancer stemness. Notably, the St → So group showed a partial reduction in these stemness markers, suggesting that stiffness‐mediated YAP activation and its downstream transcriptional programs are reversible upon mechanical relaxation.

This further highlighted the role of ECM stiffness in regulating CSC‐like behaviors (Figure 3g) [49, 50, 51, 52, 53]. Similar increases were observed in the liver cancer cell line SNU‐449 and breast cancer cell line MDA‐MB‐231 under stiff conditions (Figure S10). These results implied that ECM stiffness promoted cancer stemness through mechanotransduction, and this response was conserved across multiple cancer types.

## 4 Phenotypic Changes and Therapeutic Resistance Induced by ECM Stiffness

**FIGURE 4:**
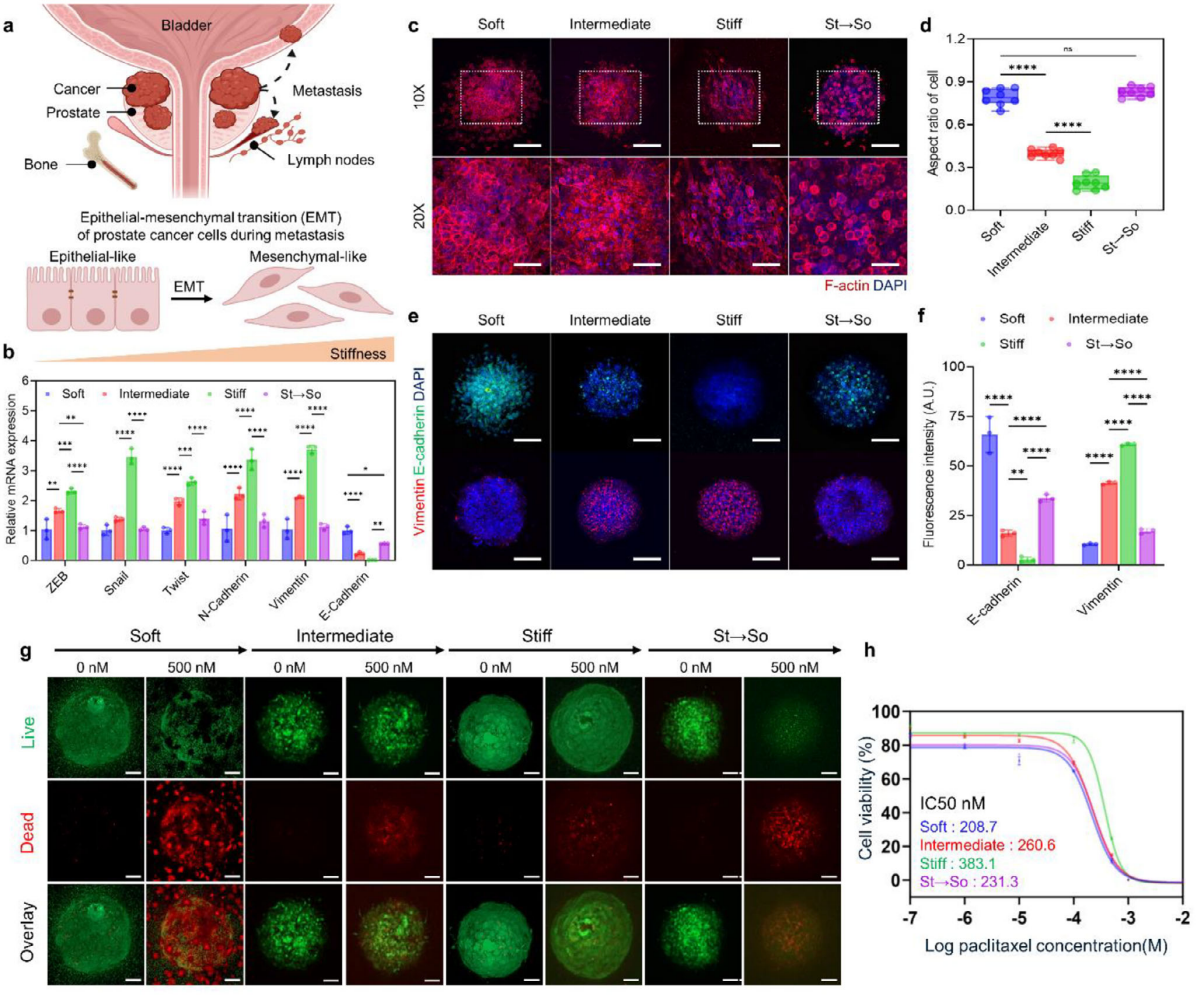
ECM stiffness promotes malignant phenotypes, including EMT and drug resistance. (a) Schematic showing prostate cancer progression and metastasis. (b) qPCR results of epithelial and mesenchymal markers in soft, intermediate, stiff and St → So groups (*n* = 3). (c) Confocal images of spheroid morphological changes in soft, intermediate, stiff and St → So groups. (upper: 10×, scale bars, 100 µm; lower: 20×, scale bars, 50 µm). (d) Quantification of aspect ratio in soft, intermediate, stiff and St → So groups (*n* = 8). (e) Confocal images of epithelial marker E‐cadherin (green, with DAPI; upper) and mesenchymal marker Vimentin (red, with DAPI; lower) in soft, intermediate, stiff and St → So groups. Scale bars, 150 µm. (f) Quantification of fluorescence intensity of E‐cadherin and Vimentin in soft, intermediate, stiff and St → So groups (*n* = 3). (g) Live/Dead staining images after 2 days of paclitaxel treatment and dimethyl sulfoxide (DMSO) control in soft, intermediate, stiff, and St → So groups. Scale bars, 100 µm. (h) Dose‐response curve for paclitaxel treatment (0.1, 1, 10, 100, 500, and 1000 nm) and DMSO control in soft, intermediate, stiff, and St → So groups, showing normalized absorbance (%) (*n* = 3). The error bars represent mean ± SD. Statistical significance was assessed using one‐way ANOVA (*p < 0.05, **p < 0.01, ***p < 0.001, ****p < 0.0001).

The impact of stiffness on EMT and drug resistance two key malignant traits linked to tumor progression and therapy resistance was investigated. These features, including stemness, EMT, and drug resistance, are known to characterize aggressive cancer phenotypes linked to metastatic potential and recurrence [54, 55, 56, 57, 58, 59, 60, 61, 62]. EMT is a critical process where epithelial cells lose cell–cell adhesion and acquire mesenchymal features, enhancing their migration and invasion. In prostate cancer, EMT significantly facilitates metastasis (Figure 4a). To investigate the effects of ECM stiffness on EMT, the expression of epithelial and mesenchymal markers under varying stiffness conditions was analyzed. Gene expression analysis revealed a significant downregulation of the epithelial marker E‐cadherin and concomitant upregulation of mesenchymal markers such as vimentin and N‐cadherin in stiffer environments, changes that were partially restored in the St → So group (Figure 4b).

Phenotypic changes associated with EMT were visualized through F‐actin staining, revealing structural reorganization due to stiffness. Cells in the stiff group exhibited elongated morphologies typical of mesenchymal phenotypes, whereas those in the soft group retained rounded, epithelial‐like shapes. Importantly, these stiffness‐induced morphological changes were reversed toward rounded forms in the St → So group (Figure 4c). Aspect ratio analysis confirmed a significant increase in cell elongation under stiffer conditions that was restored to levels comparable to the soft group upon stiffness relaxation in the St → So group (Figure 4d).

Immunofluorescence staining of E‐cadherin and Vimentin further demonstrated stiffness‐dependent EMT regulation. In the stiff group, Vimentin intensity markedly increased, whereas E‐cadherin expression decreased compared with the soft group. Importantly, these changes were reversed in the St → So group, where E‐cadherin expression was restored and Vimentin intensity was reduced to levels comparable to the soft condition (Figure 4e,f). Quantification of fluorescence intensity corroborated this reversal, confirming that stiffness‐induced EMT is dynamically regulated and partially reversible upon matrix softening.

To capture the dynamic kinetics of this phenotypic reversion, time‐course monitoring was performed in the St → So group. Time‐course analysis of the St → So transition (St_3_ → So_0_ to St_3_ → So_4_) further revealed that spheroid compaction was preserved at St_3_ → So_1_ and markedly reduced from St_3_ → So_2_ onward, accompanied by progressive loosening of spheroid architecture (Figure S11a,b). In parallel, vimentin fluorescence intensity exhibited a significant time‐dependent decrease during the stiff‐to‐soft transition (Figure S11a,c). These results illustrated the role of ECM stiffness in promoting EMT, a characteristic of malignant phenotypes. Given that ECM stiffness promotes EMT and malignant traits, we further investigated its role in enhancing drug resistance. Drug resistance is a key trait associated with aggressive cancer cell populations, allowing them to evade chemotherapy and contribute to tumor relapse [59, 60, 61, 62]. To investigate this, cell viability under chemotherapeutic treatment using live/dead staining and IC50 measurements across different stiffness conditions was examined. The live/dead assay revealed that cells in the stiff group exhibited higher survival rates, indicating enhanced drug resistance (Figure 4g). Interestingly, the St → So conditions partially restored drug sensitivity, indicating the reversible nature of this response. IC50 measurements revealed significantly higher values in the stiff group, indicating high resistance to paclitaxel in stiffer microenvironments (Figure 4h).

To investigate the correlation between drug resistance and baseline proliferation rates, cell growth was analyzed across various stiffness conditions (Figure S12a). Under normal culture conditions, cells in the soft group exhibited the highest proliferation rates, with rates declining as stiffness increased. This trend aligned with the mechanism of primary chemotherapeutic agents such as paclitaxel, which predominantly target actively proliferating cells [63, 64, 65]. Supporting this finding, immunofluorescence staining of Ki‐67 revealed a marked reduction in proliferative activity with increasing stiffness, consistent with the quantitative growth assay results. In contrast, cleaved Caspase‐3 staining showed negligible expression across all groups, confirming that the observed differences in proliferative patterns were not attributable to apoptosis (Figure S12b). The lower proliferation rates in the stiff group suggest that additional mechanisms contribute to their drug resistance. Importantly, proliferation dynamics under paclitaxel treatment revealed that growth was reduced across all groups, yet the degree of suppression varied with matrix stiffness. The stiff condition exhibited the least reduction, consistent with a relative insensitivity to paclitaxel, whereas the St → So group showed a marked reduction compared with stiff, indicating a partial restoration of chemosensitivity upon stiffness reversal (Figure S13) [66]. This pattern likely reflects key characteristics of CSC‐like cells, which maintain a quiescent state prioritizing self‐renewal over rapid proliferation. Quiescence acts as a protective mechanism, enabling CSCs to evade the effects of chemotherapeutic agents that target dividing cells. This state supports the long‐term maintenance of stemness and the regenerative capacity of CSCs, leading to tumor recurrence and therapy resistance. Collectively, these results indicate that increased ECM stiffness modulates baseline proliferative activity, and that the resulting reduction in proliferation contributes to decreased sensitivity to mitotic inhibitors such as paclitaxel.

## 5 Transcriptomic Profiling of Stiffness‐Induced Reprogramming and Therapeutic Targets

**FIGURE 5:**
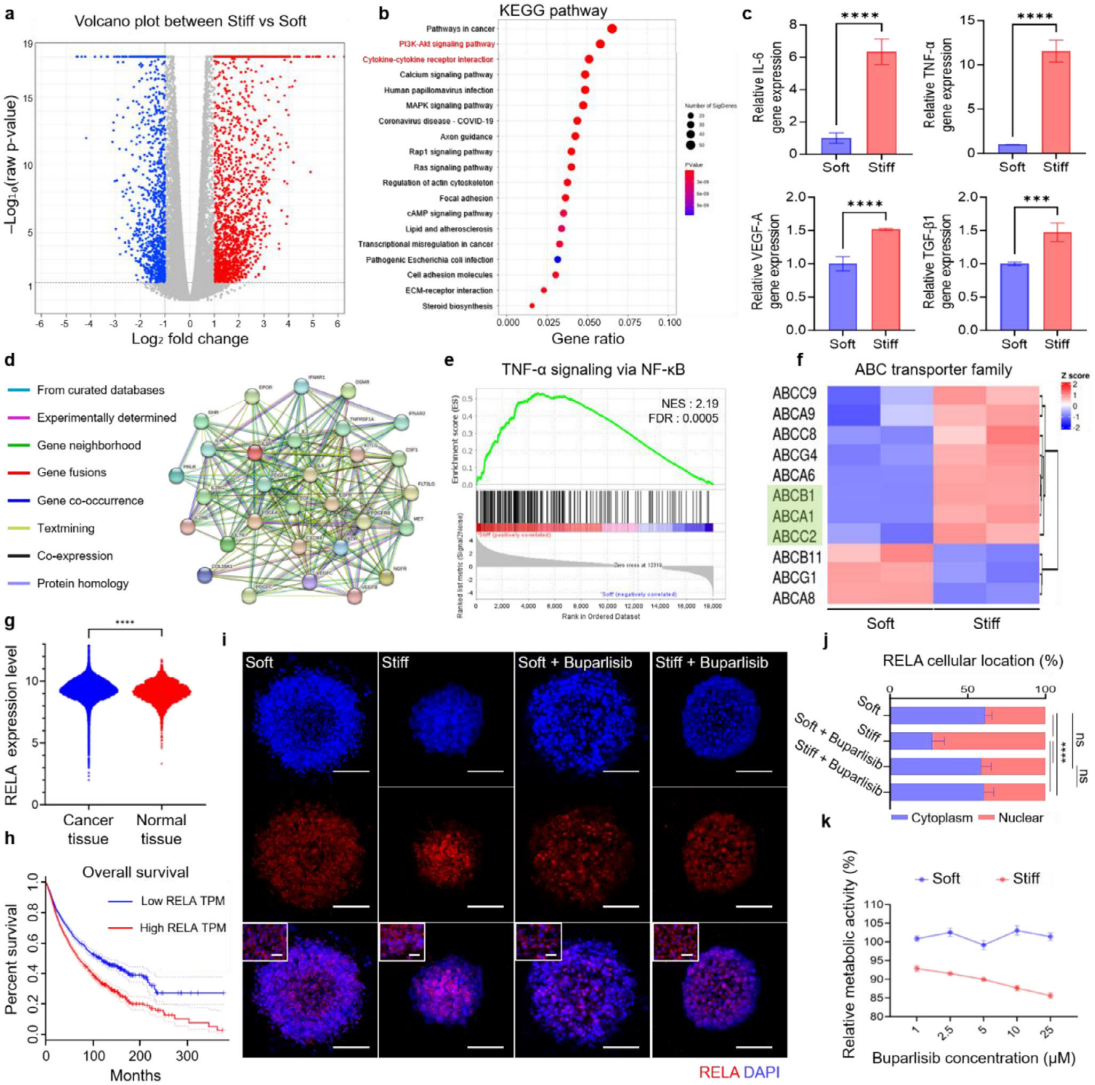
Activation of PI3K/NF‐κB signaling by ECM stiffness. (a) Volcano plot comparing gene expression between Soft and Stiff groups. (b) KEGG pathway enrichment analysis of genes with increased expression in PC3 cells cultured in stiff group compared with soft group. (c) Quantification of gene expression for major cytokines in cytokine–cytokine receptor interaction category (*n* = 3). (d) STRING network analysis of overlapping genes between PI3K/Akt signaling and cytokine–cytokine receptor interaction pathways (e) GSEA plot showing NF‐κB signaling as the top enriched pathway under stiff conditions (NES = 2.19, FDR = 0.0005). (f) Heatmap analysis of ABC transporter expression in soft and stiff groups. (g) Database‐based comparison of RELA expression levels between normal and prostate cancer tissues. (h) Kaplan–Meier survival analysis stratified by RELA expression showing overall survival. (i) Confocal images of RELA and DAPI staining in soft, stiff, soft + buparlisib, and stiff + buparlisib groups. Enlarged views of nuclear regions are shown with scale bars of 50 µm; full‐field images have scale bars of 200 µm. (j) Quantification of RELA nuclear translocation in soft, stiff, soft + buparlisib, and stiff + buparlisib groups (*n* = 3). (k) Dose‐dependent comparison of relative metabolic activity between soft and stiff groups following buparlisib treatment. Values were normalized to DMSO‐treated controls (*n* = 3). The error bars represent mean ± SD. Statistical significance was assessed using one‐way ANOVA (*p < 0.05, **p < 0.01, ***p < 0.001, ****p < 0.0001).

A system with tunable stiffness was developed to explore transcriptional reprogramming linked to malignant progression. This platform shows promise for modeling stiffness‐induced malignant phenotypes and drug screening in cancer cells resistant to therapies. RNA sequencing was conducted on PC3 cells cultured under soft and stiff conditions. Hierarchical clustering of differentially expressed genes (DEGs) revealed distinct transcriptional profiles between the groups, highlighting stiffness‐induced reprogramming (Figure 5a). A total of 3,535 significantly modulated genes were identified (fold change ≥ 2, *p*‐value < 0.05), with 2,169 genes upregulated and 1,366 genes downregulated in the stiff group (Figure S14). To evaluate the clinical relevance of the stiffness‐driven malignant model, we defined a survival‐associated gene signature by selecting the top 100 genes whose higher expression significantly correlated with poor overall survival in prostate cancer patients, based on TCGA and GTEx datasets. Principal component analysis of patient samples confirmed that this signature clearly distinguished tumor from normal tissues (Figure S15a).

Building on this, we evaluated the representation of the survival‐associated gene set in our transcriptomic dataset and found that 77 of these genes were upregulated in the stiff group compared with the soft group, indicating that stiffness‐induced transcriptional reprogramming in our model shares similarities with adverse patient‐associated signatures (Figure S15b). Subsequently, we sought to identify the molecular pathways underlying this stiffness‐driven malignant shift. KEGG pathway enrichment analysis revealed that PI3K/Akt signaling, cytokine–cytokine receptor interaction, and calcium signaling pathways were highly significant, all known to promote stemness, drug resistance, and EMT (Figure 5b) [67, 68, 69, 70, 71, 72, 73]. GSEA analysis confirmed the enrichment of multiple signaling pathways linked to malignant progression, including EMT (NES = 1.59, FDR = 0.01), hedgehog signaling (NES = 1.68, FDR = 0.006), TGF‐β signaling (NES = 1.63, FDR = 0.006), and JAK–STAT signaling (NES = 1.47, FDR = 0.01). This indicated a broader activation of stiffness‐responsive oncogenic programs (Figure S16) [74, 75, 76, 77]. To elucidate the molecular basis of stiffness‐induced transcriptional reprogramming, the expression of key cytokines related to cytokine–cytokine receptor interaction pathway was examined, which was notably enriched in the KEGG analysis. In the stiff group, there was a significant upregulation of TNF, IL‐6, VEGF‐A, and IL‐1β (Figure 5c). This upregulation was likely driven by increased hypoxia and the activation of key signaling pathways, suggesting that ECM stiffness enhanced cytokine signaling by modulating TME factors [5, 78, 79]. Given that the KEGG analysis ranked PI3K/Akt and cytokine–cytokine receptor interaction among the top pathways, these pathways were hypothesized not to act independently but exhibit strong functional interconnectivity. To further investigate the relationship between PI3K/Akt and cytokine–cytokine receptor interaction, STRING network analysis focused on key components revealed significant molecular interactions between shared genes. This indicated that these pathways were functionally interconnected (Figure 5d). GSEA analysis identified the NF‐κB signaling pathway as having the highest normalized enrichment score (NES = 2.19, FDR < 0.0005), highlighting its prominent involvement in the stiffness‐induced cellular response (Figure 5e). The inflammatory response pathway, closely associated with NF‐κB activation, showed significant enrichment (NES = 2.01, FDR < 0.004) (Figure S16) [80, 81]. This further supported the involvement of NF‐κB–associated inflammatory programs in the stiffness‐induced response. NF‐κB is an established downstream effector of PI3K‐mediated signaling [82, 83, 84] and a key regulator activated by cytokine signaling and stiffness‐related mechanical cues [85, 86, 87]. To further validate this signaling model, the expression of ABC transporters was analyzed, which are established downstream targets of NF‐κB that develop chemoresistance [88, 89, 90]. ABCB1 (FC = 4.39), ABCA1 (FC = 3.02), and ABCC2 (FC = 3.36) were significantly upregulated in the stiff group, with ABCB1 exhibiting the highest fold change (Figure 5f). These transporters actively effluxe chemotherapeutic agents from the cell, reducing intracellular drug accumulation and enhancing treatment resistance [91, 92, 93]. This upregulation of ABC transporters aligned with the high level of drug resistance previously observed in the stiff group (Figure 4g), reinforcing the notion that ECM stiffness enhanced chemo‐resistant phenotypes through NF‐κB mediated mechanisms. In addition, expression profiling across the cytokine and integrin‐mediated PI3K/NF‐κB axis under stiff conditions revealed a coordinated cascade‐level activation (Figure S17a,b). Upstream regulators that activate PI3K, including cytokine receptors (IL6R, TNFRSF1A, TGFBR1/2), integrin receptors (ITGB1, ITGA5, ITGA6), and components of the integrin–FAK module (TLN1, FERMT2, PTK2, SRC), were markedly upregulated under stiff conditions, indicating that matrix stiffness activates both biochemical and mechanical pathways converging on PI3K. The negative regulator PTEN was suppressed, accompanied by increased expression of PI3K subunits (PIK3CA, PIK3CB, PIK3CD, PIK3R1, PIK3R3/4) and downstream kinases (AKT1/2), followed by elevated levels of NF‐κB transcription factors (RELA, NFKB1) and effector genes (BCL2L1, MYB). Collectively, these findings demonstrate that matrix stiffness enhances PI3K activation through both cytokine‐receptor and integrin–FAK modules, which converge to amplify NF‐κB‐dependent transcriptional programs [94, 95, 96, 97, 98]. This was consistent with reports on the role of ECM stiffness in regulating intracellular signaling dynamics and pathway interconnectivity [82, 83, 84, 85, 86, 87]. To evaluate the clinical relevance of the transcriptional reprogramming observed in the stiffness‐modulated system, RELA expression and patient survival were analyzed using large‐scale cancer datasets. RELA was significantly upregulated in tumor tissues compared with normal tissues (*p* < 0.0001), with patients exhibiting high RELA expression showing reduced overall survival (Log–rank test, *p* = 0.0336) (Figure 5g,h). These findings highlighted clinical association with the NF‐κB‐driven transcriptional program identified in the model, reinforcing the pathological relevance of stiffness‐induced malignant phenotypes. Nuclear translocation of RELA a key transcription factor in the NF‐κB signaling pathway was assessed in soft and stiff groups. The PI3K inhibitor buparlisib was selected as a targeted therapeutic agent, given the role of PI3K as a key regulator within this signaling cascade [99, 100]. RELA nuclear translocation significantly increased in the stiff group, indicating enhanced NF‐κB activation under stiff conditions. Buparlisib treatment effectively suppressed RELA nuclear translocation in the stiff group, comparable to the soft group. In contrast, buparlisib had no significant effect on RELA localization in the soft group (Figure 5i,j).

Increased nuclear translocation of NF‐κB in the stiff group is predominantly mediated by PI3K signaling, supporting the hypothesis. To evaluate the therapeutic potential of PI3K inhibition, the metabolic response to buparlisib treatment was examined across a range of concentrations. No significant changes occurred in the soft group, whereas buparlisib treatment caused a dose‐dependent reduction in metabolic activity in the stiff group. This suggested that PI3K inhibition selectively affected cells with stiffness‐induced therapeutic resistance (Figure 5k). Stiffness‐induced resistant cells exhibited elevated reliance on PI3K signaling for metabolic activity, making them more susceptible to pathway‐specific inhibition.

## 6 Stiffness‐Induced Resistance is Reversed by PI3K Inhibition, Restoring Chemosensitivity and Suppressing Invasion

**FIGURE 6:**
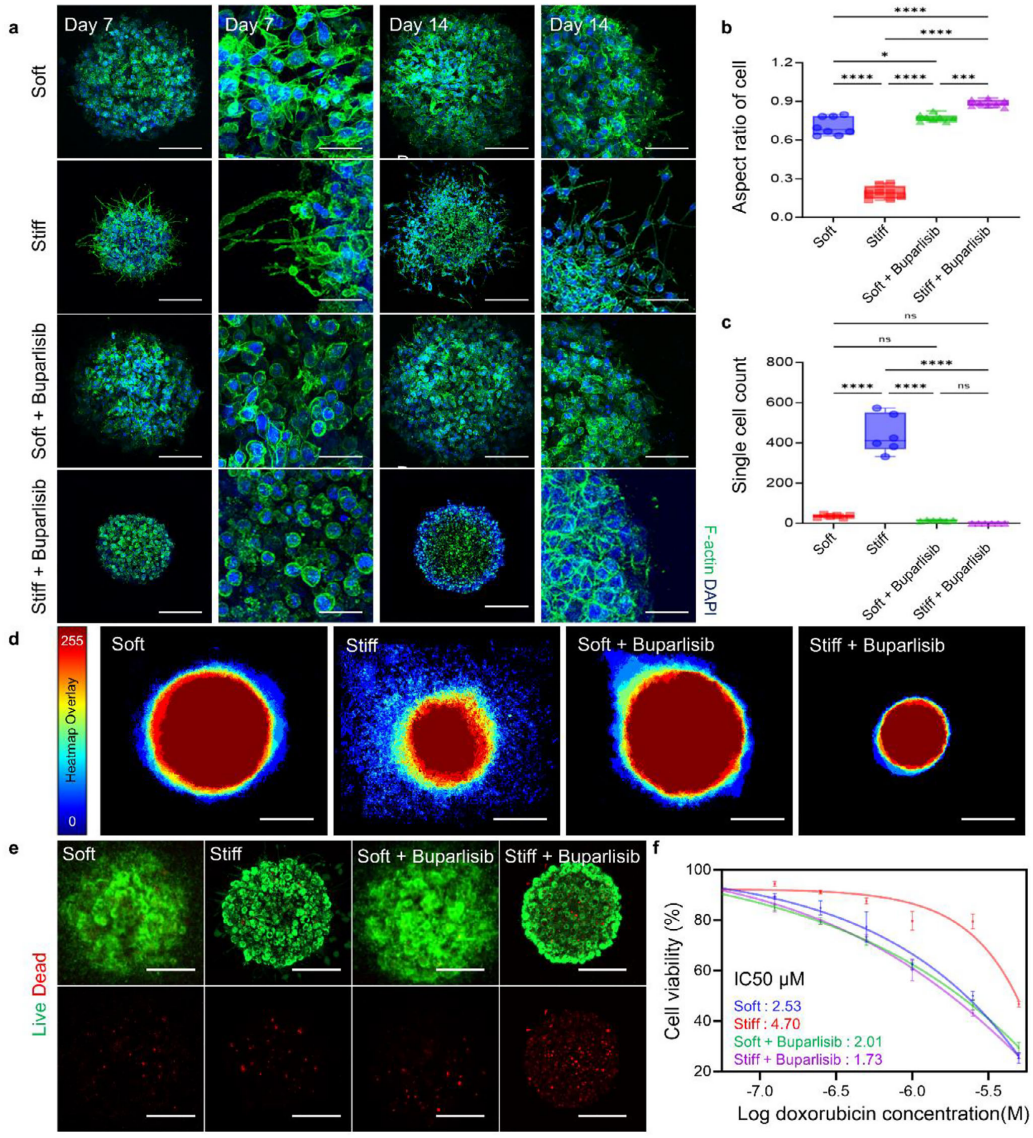
Assessment of PI3K‐targeted therapy in a stiffness‐induced resistant cancer cells. (a) Confocal images of F‐actin and DAPI staining at days 7 and 14 in soft, stiff, soft + buparlisib, and stiff + buparlisib groups. Scale bars, 200 µm (day 7), 50 µm (enlarged, day 7), 200 µm (day 14), and 100 µm (enlarged, day 14). (b) Quantification of aspect ratio in soft, stiff, + buparlisib, and stiff + buparlisib groups (*n* = 8). (c) Quantification of single‐cell counts in soft, stiff, soft + buparlisib, and stiff + buparlisib groups. Single cells are defined as individual units observed in postimaging analysis where no cell overlap is present (*n* = 6). (d) Spatial heatmaps are generated by aligning and overlaying 15 individual spheroids per condition to evaluate drug‐induced responses under different stiffness environments. Scale bars, 300 µm. (e) Live/Dead staining images following 25 µm buparlisib treatment in soft, stiff, + buparlisib, and stiff + buparlisib groups. Scale bars, 200 µm. (f) Dose‐response curve for doxorubicin treatment (0.05, 0.125, 0.25, 0.5, 1, 2.5, 5 µm) and DMSO control in soft, stiff, soft + buparlisib, and stiff + buparlisib groups, showing normalized absorbance (%) (*n* = 3). The error bars represent mean ± SD. Statistical significance was assessed using one‐way ANOVA (*p < 0.05, **p < 0.01, ***p < 0.001, ****p < 0.0001).

Mechanistic analysis identified PI3K as a central regulatory node within the stiffness‐induced signaling cascade and selected buparlisib as a targeted therapeutic agent. To assess the functional relevance of this therapeutic strategy, the effect of PI3K inhibition on enhanced EMT and drug resistance phenotypes under stiff conditions was investigated. On day 7, cells in the stiff group maintained the elongated, high aspect ratio morphology of a mesenchymal phenotype. In contrast, cells in the stiff + buparlisib group exhibited a more rounded, epithelial‐like morphology (Figure 6a,b), indicating a mesenchymal‐to‐epithelial transition (MET) induced by buparlisib treatment. The soft + buparlisib group showed no notable changes in cell morphology compared with the untreated soft group.

Building on morphological observations of day 7, the culture period was extended to 14 days to examine stiffness‐dependent migration patterns and evaluate the effect of PI3K inhibition on invasive behavior. The soft and soft + buparlisib groups exhibited collective migration, typically observed in compliant microenvironments [101, 102, 103]. In the stiff group, single‐cell migration was observed at the periphery, indicating invasive phenotype (Figure 6c). This phenotype was absent in the stiff + buparlisib group, demonstrating that PI3K inhibition effectively suppressed stiffness‐induced migratory plasticity. Composite fluorescence projections of multiple spheroids (*n* = 15) under each condition revealed consistent spatial patterns of migration and drug response (Figure 6d). To further investigate the effect of PI3K inhibition on cell viability, live/dead staining was performed following buparlisib treatment in the soft, soft + buparlisib, stiff, and stiff + buparlisib groups. Consistent with the metabolic activity results, an increase in dead cell signal was observed in the stiff + buparlisib group, whereas no changes were detected in the soft + buparlisib group (Figure 6e) (Figure S18). This suggested that PI3K inhibition minimally impacted soft conditions but induced cell death in stiffness‐induced, therapeutically resistant subpopulations. The strong association between PI3K/NF‐κB signaling and survival pathways supported that stiffness‐mediated reprogramming enhanced cellular reliance on this axis for survival and evasion of apoptosis [82, 83, 84].

To determine whether PI3K inhibition enhanced the efficacy of standard chemotherapy, the response of buparlisib‐pretreated cells to increasing concentrations of doxorubicin a widely used chemotherapeutic agent was evaluated. Unlike paclitaxel, which primarily targets mitotic progression, doxorubicin induces cytotoxicity through DNA intercalation, topoisomerase II inhibition, and ROS generation. The use of doxorubicin therefore enabled us to evaluate whether matrix‐induced quiescence and stemness traits confer resistance beyond mitotic inhibitors, extending to DNA‐damaging agents as well [104]. Consistent with the previous results demonstrating substantial resistance to paclitaxel in the stiff group, these cells exhibited markedly increased resistance to doxorubicin compared to other groups. The pronounced resistance observed in the stiff group suggests that matrix‐induced quiescence and stemness traits may attenuate the effects of mitotic inhibitors such as paclitaxel, as well as those of doxorubicin, which relies on both active DNA replication and DNA damage induction. These results confirm the broad‐spectrum chemoresistant nature intrinsically acquired by cells in stiff environments [59, 60, 61, 62]. Taken together, these findings demonstrate that ECM stiffness contributes to drug resistance through a multifaceted mechanism. The acquisition of quiescent and stemness‐associated traits under stiff conditions serves as the primary driver, giving rise to an intrinsic and broad‐spectrum chemoresistant phenotype that extends beyond mitotic inhibitors to DNA‐damaging agents such as doxorubicin. In addition, stiffness‐induced suppression of baseline proliferative activity functions as a secondary contributor that specifically reduces sensitivity to mitosis‐targeting drugs like paclitaxel.

This emphasizes the critical need for therapeutic strategies targeting specific mechanisms to counteract stiffness‐mediated drug resistance and improve clinical outcomes. Importantly, PI3K inhibition, identified through molecular profiling using the stiffness‐modulated system, effectively restored chemosensitivity in the stiff group, reducing drug resistance and enhancing the cytotoxic response to doxorubicin (Figure 6f). In parallel, proliferation assays confirmed that combined buparlisib and doxorubicin treatment produced the most pronounced suppression in the stiff group, indicating that stiffness‐induced resistance is maintained by PI3K/NF‐κB‐driven survival pathways, such as the anti‐apoptotic mediator BCL2L1 and drug‐efflux ABC transporters, and can be effectively reversed through PI3K inhibition (Figure S19) [105]. This highlights the utility of the stiffness‐modulated system as a potential strategy to overcome stiffness‐driven treatment resistance [67].

## 7 Conclusion

This study developed a bespoke hydrogel with tunable stiffness by incorporating dECM and alginate to mimic biomechanical features of the prostate TME. Using in‐bath 3D bioprinting, uniformly sized cancer spheroids were generated within this engineered hydrogel, effectively modeling stiffness‐dependent tumor behavior. Increased mechanical stress led to enhanced integrin signaling, EMT, and chemoresistance of the spheroids. NGS analysis identified that the PI3K signaling pathway, an upstream regulator of NF‐κB, was most responsive under stiff conditions. Inhibition of PI3K markedly attenuated NF‐κB nuclear translocation induced by mechanical stress. Furthermore, cancer aggressiveness and chemoresistance under stiff conditions were significantly reversed to levels comparable to those in soft conditions with minimal mechanical stimulation.

These findings suggest that the developed platform holds significant potential for elucidating the mechanisms of prostate cancer progression driven by mechanotransduction, and for developing effective therapeutic strategies targeting these pathways. To extend its applicability to a broader spectrum of cancers and enhance translational relevance, further investigations are warranted:

- To accurately recapitulate the patient‐specific TME and variability in drug responses, future studies should develop personalized cancer models using patient‐derived tumor organoids.
- Although recent advances have led to the active development of high‐throughput screening platforms using microfluidic, challenges in mass production and precise spheroid positioning limit their use. The in‐bath 3D bioprinting technique presented in this study offers a distinct advantage in generating large quantities of uniformly sized spheroids, suitable for integration with microfluidic platforms, for scalable anticancer drug screening.
- Considering the critical role of TME heterogeneity in oncogenesis, incorporating key stromal, immune, and vascular cell populations may allow for a more physiologically relevant reconstruction of the TME in vitro, thereby providing deeper insights into the interplay between matrix stiffness and tumor heterogeneity.
- To enhance the physiological relevance and clinical applicability of the platform, future studies will focus on incorporating biochemical heterogeneity specific to the prostate tumor microenvironment together with stiffness modulation to more faithfully represent patient‐specific tumor microenvironments.
- While this study established stiffness ranges based on clinical literature, the ultimate goal is to develop truly patient‐specific models constructed from direct mechanical measurements of individual patient tissues. By incorporating patient‐derived mechanical data obtained through techniques such as shear‐wave elastography or atomic force microscopy, future models could precisely replicate the unique biomechanical signatures of each patient's tumor microenvironment, enabling personalized mechanobiological profiling and therapeutic optimization.

Building upon these future directions, subsequent studies will further broaden the applicability of this platform across diverse cancer types and enhance its translational relevance.

In conclusion, this study demonstrates that in‐bath 3D bioprinting, combined with a stiffness‐tunable hydrogel, enables the investigation of how biophysical cues promote prostate cancer progression via the PI3K/NF‐κB signaling axis. Moreover, pharmacological inhibition of PI3K effectively suppresses cancer malignancy, highlighting PI3K as a promising therapeutic target for prostate cancer. The developed platform offers potential for advancing precision oncology and designing targeted therapies for prostate cancer.

## 8 Methods

### 8.1 Cancer Cell Culture

Human cancer cell lines PC‐3 (RRID: CVCL_0035) and MDA‐MB‐231 (RRID: CVCL_0062) were purchased from ATCC. The human cancer cell line SNU‐449 (RRID: CVCL_0454) was obtained from the Korean Cell Line Bank (KCLB, Seoul, Republic of Korea). All cell lines were stored according to the supplier's instructions and used within 6 months after resuscitation of frozen aliquots. Cells were maintained in RPMI‐1640 medium (Gibco) supplemented with 10% fetal bovine serum (FBS, Gibco) and 1% penicillin–streptomycin (Pen‐Strep, Gibco). For subculturing, cells grown in 2D culture dishes were dissociated with 0.25% trypsin‐EDTA (Sigma‐Aldrich) and resuspended in fresh medium every 2–3 days. All cell lines were routinely tested for mycoplasma contamination using the MycoAlert Mycoplasma Detection Kit (LT07‐318, Lonza) and confirmed to be negative.

### 8.2 Hybrid Bioink Formation

All hybrid bioinks contained 1.0% w/v dECM and ≈0%–1% wt/vol low‐viscosity alginate (Sigma‐Aldrich). Sodium alginate stock solutions (5% wt/vol; Sigma‐Aldrich) were prepared by dissolving alginate in distilled water with overnight stirring. Alginate concentrations of 1%, 0.75%, 0.5%, 0.25%, and 0% were used for to generate stiffness‐controlled hybrid bioinks, mixed with dECM while on ice to prevent pregelation. Prostate cell suspensions were also cooled on ice and mixed to achieve a total of 1 × 10^8^ cells per ml of hybrid bioink solution. The dECM and alginate mixture was formed into a PEVA structure. The dECM was allowed to gel at 37 °C for 1 h, after which a 100 mm calcium chloride (CaCl_2_; Sigma‐Aldrich) solution was added to cross‐link the alginate through diffusion of Ca^2+^ ions for 1 h at 37 °C. finally, the mixture was washed 3 times with cell culture media.

### 8.3 Characterization of Hybrid Bioink

SEM images were obtained using a Zeiss Gemini 500 scanning electron microscope. The hybrid bioink underwent thermal cross‐linking for 40 min, ionic cross‐linking for 40 min, and freeze‐drying at −20 °C for 24 h. Cross‐sections of the bioink were imaged at an accelerating voltage of 5 kV. Porosity and pore wall thickness were analyzed using ImageJ. The swelling ratio was determined by comparing the weight of the bioink before and after lyophilization and immersion in phosphate‐buffered saline (PBS) for 24 h.

### 8.4 Evaluation of Rheological Properties of Hybrid Bioink to Apply In‐Bath Bioprinting

Rheological properties of the hybrid bioink were measured using a 25 mm parallel‐plate rheometer (TA Instruments). Storage and loss moduli were determined through strain sweeps from 0.01% to 100%. The shear recovery behavior of samples with varying concentrations was assessed by varying the shear strain from 1% and 100% at a constant temperature of 15 °C. Shear thinning effect were evaluated with steady shear sweep analyses at the same temperature.

### 8.5 IF Staining

For immunostaining, samples were fixed with 4% w/v paraformaldehyde solution and permeabilized with 0.1% Triton X–100 solution (Biosesang). To minimize background, they were and treated with 1% w/v bovine serum albumin (Life Science). Primary antibodies were incubated overnight at 4 °C followed by washes with 1 × PBS. Secondary antibodies, Alexa Fluor 488 and 594, were added and incubated for 2 h at room temperature. Samples were counterstained with DAPI (Thermo Fisher Scientific) for 1 h and imaged using a confocal microscope (LSM 900, Zeiss).

### 8.6 mRNA Expression Analysis

To analyze mRNA expression in prostate cancer cells within hybrid bioink, RNAs were extracted using the TRIzol reagent (Ambion) and chloroform (Sigma‐Aldrich) via the phenol/chloroform method. The extracted mRNA was precipitated using isopropyl alcohol (IPA; Sigma‐Aldrich) and glycogen (Roche) and washed with 75% ethanol in diethylpyrocarbonate‐treated water (Biosesang). After dehydration, the RNA was dissolved in diethylpyrocarbonate‐treated water (Biosesang). The quantity and purity of the total RNA were measured using a nanodrop spectrophotometer (Thermo). The purified RNA was reverse transcribed to complementary DNA using an iScript cDNA Synthesis Kit (Bio‐Rad). Gene expression was quantitatively detected with SYBRgreen using LightCycler 480 System (Hoffmann‐La Roche). Primers were designed based on published gene sequences from NCBI.

### 8.7 In‐Bath Bioprinting of Prostate Spheroids

Porcine skin tissue from a slaughterhouse (Majang‐dong) was decellularized and formulated into a bioink as described in prior studies [8, 9, 10, 11]. Lyophilized tissues were dissolved in 0.5 m CH_3_COOH solution containing 10 mg of pepsin per 100 mg of dECM for seven days. After the complete dissolution of dECM, the pH was adjusted to 7.4 using a 10 m NaOH solution. PC3 were encapsulated in 1% w/v dECM at a density of 1 × 10^8^ cells per mL and loaded in a sterile syringe with a 26 G stainless steel microneedle. Each hybrid bioink group was placed in a PEVA (PolyScience) structure. Prostate spheroids were printed within the hybrid bath for 200 ms at a pressure of 3–6 kPa along a predefined path. The printing pressure was varied to adjust the spheroid diameter. After printing, the needle was withdrawn, and the tissues were incubated at 37 °C for cross‐linking.

### 8.8 Cell Viability Assay

The viability of PC3 after encapsulation and printing was assessed using a live/dead viability kit (Invitrogen). All groups were incubated at 37 °C for thermal cross‐linking. The hybrid bioink with alginate underwent an additional 40 min incubation at 37 °C in 100 mm CaCl_2_ solution for ionic cross‐linking. The group without alginate was incubated under the same conditions. After washing with PBS, all groups were stained with calcein AM and ethidium homodimer (EthD‐1) in PBS. The cell viability was evaluated by calculating the ratio of viable cells to the total cell number under a fluorescence microscope (Axio Zoom, Zeiss).

### 8.9 Image Analysis

Nuclear YAP and RELA localization was analyzed using Image J/FIJI (64‐bit Java 1.8.0). Cytoplasmic YAP intensity was measured by subtracting the nuclear (DAPI) intensity from the total YAP intensity. Nuclear YAP intensity was recorded as the proportion of total YAP intensity that overlapped with the nucleus (DAPI).

### 8.10 Morphometric Analysis of Cancer Spheroids

Fluorescence images of prostate cancer spheroids were captured using an Axio Zoom microscope (Zeiss) at various times. Three gels were analyzed per group. The diameter, area, and solidity of cancer cells printed or encapsulated in hybrid bioink of various stiffnesses were assessed using ImageJ. Statistical analysis was conducted on ten images from three biologically independent experiments, with solidity calculated using the formula: area / convex area.

### 8.11 Drug Resistance Analysis

A total of 1 × 10^8^ cells per mL spheroids were printed in hybrid bioink and cultured for 7 days to stimulate stemness. After removing the medium, the spheroids were washed with PBS and treated with paclitaxel at each concentration for 2 days. The samples were then transferred to culture media containing CCK‐8 solution and incubated for 3 h. Finally, the culture media were collected, and the absorbance was measured at 450 nm using a microplate reader.

### 8.12 Statistical Analysis

Statistical analysis was conducted using Prism version 8 (GraphPad). Comparisons between two groups were made using an unpaired two‐tailed *t*‐test. ANOVA was used for multiple group comparisons. *p* values are represented as asterisks on graphs (*p < 0.05; **p < 0.01; ***p < 0.001; ****p < 0.0001). All experimental values represent a minimum of three individual experiments.

## Conflicts of Interest

The authors declare no conflicts of interest.

## Supporting information




**Supporting File 1**: advs73661‐sup‐0001‐SuppMat.docx.

**Supporting File 2**: advs73661‐sup‐0002‐Videos.zip.

## Data Availability

The data that support the findings of this study are available from the corresponding author upon reasonable request.
