# Supplementary material for: Mechanical Stimuli‐Induced Manipulation of Malignant Behavior in Bioprinted Cancer Microtissues via PI3K/NF‐κB Activation
Source: Adv Sci (Weinh). 2026 Jan 4;13(16):e18295. doi: 10.1002/advs.202518295 (PMC13042440; doi:10.1002/advs.202518295)
Supplement: Supplementary file 1 — Supporting File 1: advs73661‐sup‐0001‐SuppMat.docx. [file ADVS-13-e18295-s001.docx]

**Supporting Information for**

Mechanical Stimuli-Induced Manipulation of Malignant Behavior in Bioprinted Cancer Microtissues via PI3K/NF-κB Activation

Seok-Hyeon Lee^a^, Jeongho Lee^a^, Min-Seo Choi^a^, Minjun Ahn^b,^ Sik Yoon^c^, Dongjun Lee^d,e^, Sae-Ock Oh^c^, Won-Woo Cho^f*^, Byoung Soo Kim^a,b,g*^

*^a^* *School of Biomedical Convergence Engineering, Pusan National University, Yangsan, Republic of Korea*

*^b^* *Medical Research Institute, Pusan National University, Yangsan, 50612 Republic of Korea*

*^c^ Department of Anatomy, School of Medicine, Pusan National University, Yangsan 50612, Republic of Korea*

*^d^ Department of Convergence Medicine, School of Medicine, Pusan National University, Yangsan 50612, Republic of Korea*

*^e^ Transplantation Research Center, Research Institute for Convergence of Biomedical Science and Technology, Pusan National University Yangsan Hospital, Yangsan 50612, Republic of Korea*

*^f^* *Department of Biomedical Engineering, Yonsei University, Wonju, Republic of Korea*

*^g^* *Research Institute for Convergence of Biomedical Science and Technology, Pusan National University Yangsan Hospital, Yangsan, Republic of Korea*

* Corresponding authors

E–mail: ww.cho@yonsei.ac.kr, bskim7@pusan.ac.kr

Extended Data Display/ Supplementary Information

**
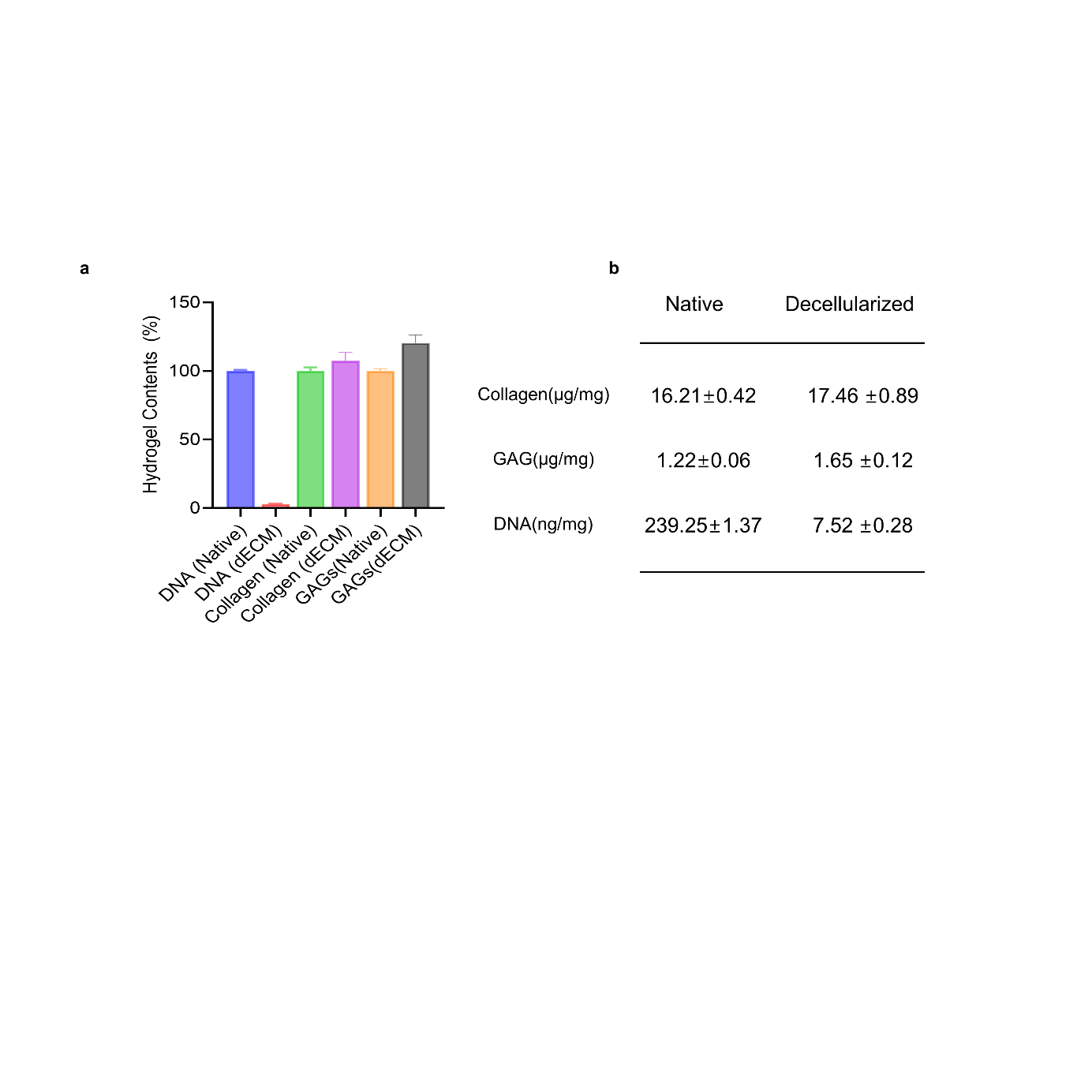
**

**Supplementary Fig. 1 | Quantitative comparison of ECM components between decellularized and native tissue samples.** (a) Relative differences in collage, glycosaminoglycans, and DNA content between native and dECM. Decellularization significantly reduces DNA content, while retaining substantial amounts of collagen and GAGs, essential for bioactive properties of dECM (n = 3). (b) Table presents quantitative comparison of collagen (µg/mg), glycosaminoglycans (GAGs, ng/mg), and DNA content (ng/mg) in dECM compared with native tissue samples. Data are presented as mean ± SD

**
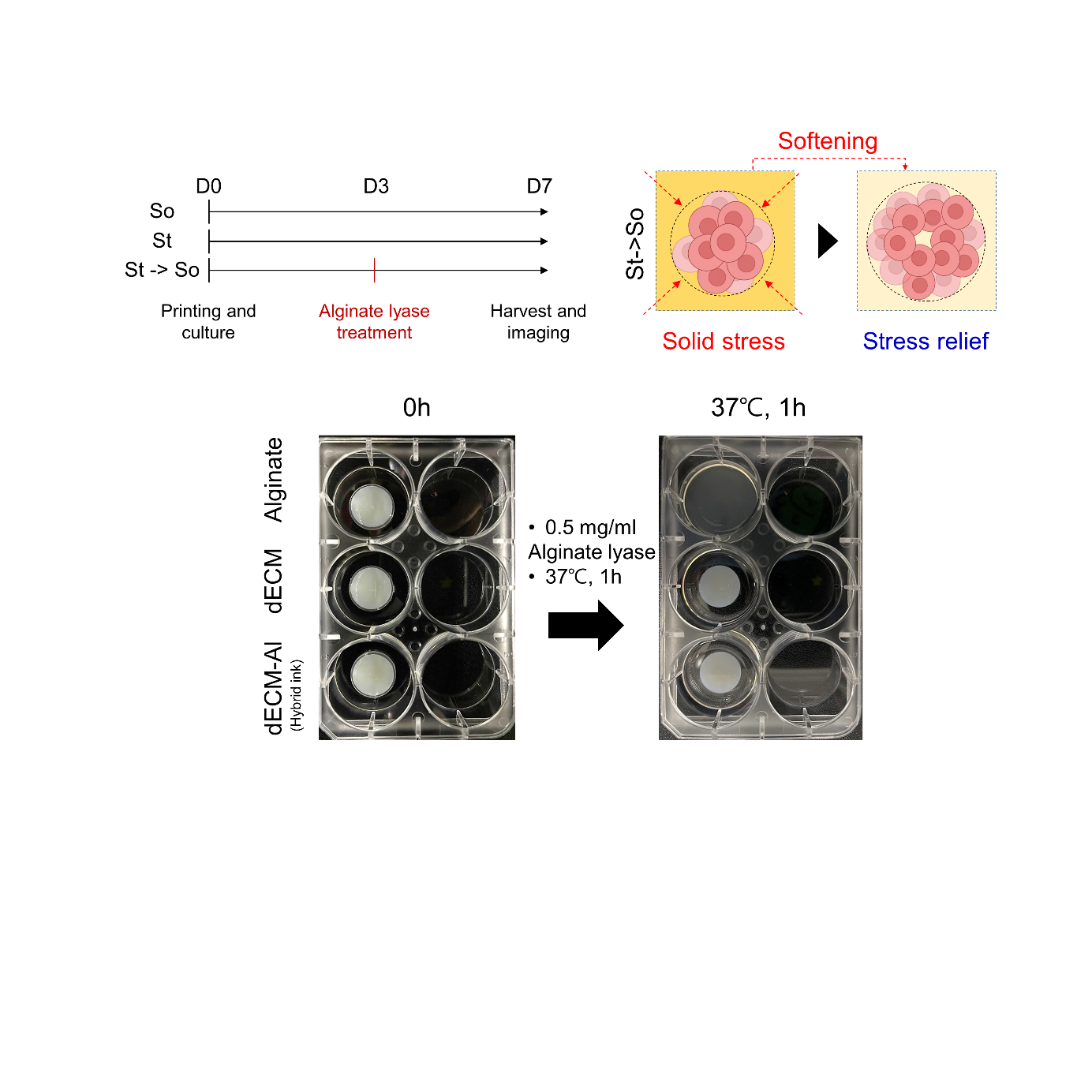
**

**Supplementary Fig. 2 | Design of the stiff → soft condition.** Stiff group is treated with 0.5 mg of alginate lyase for 1 h on day 3 to selectively degrade stiffness-controlling alginate component. This treatment is used to relieve solid stress exerted on spheroids under stiff conditions, generating the St → So group.

**
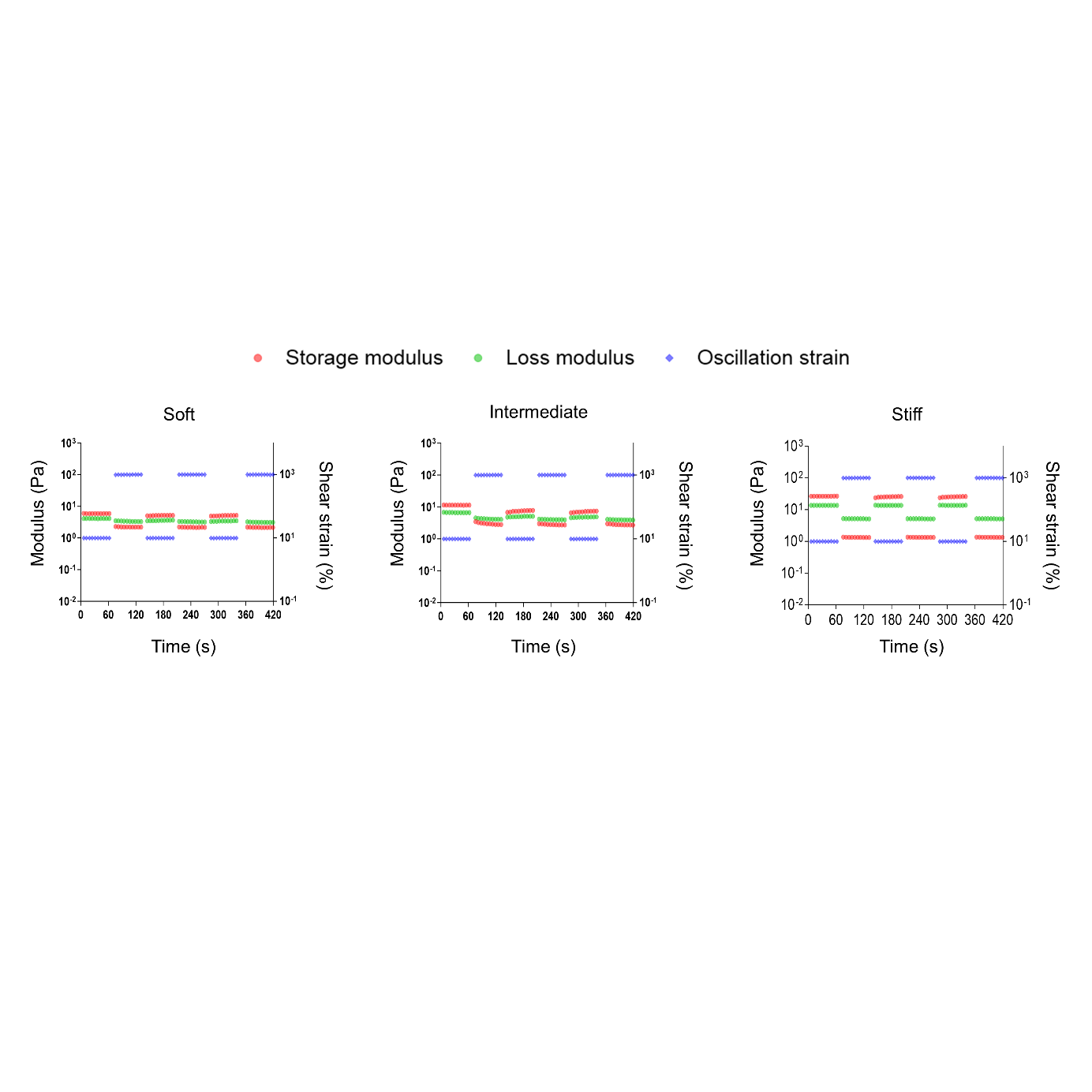
**

**Supplementary Fig. 3 | Printability evaluation of the hybrid bioink.** Shear recovery properties are evaluated by measuring storage modulus and loss modulus of hybrid bioink under varying strain conditions.


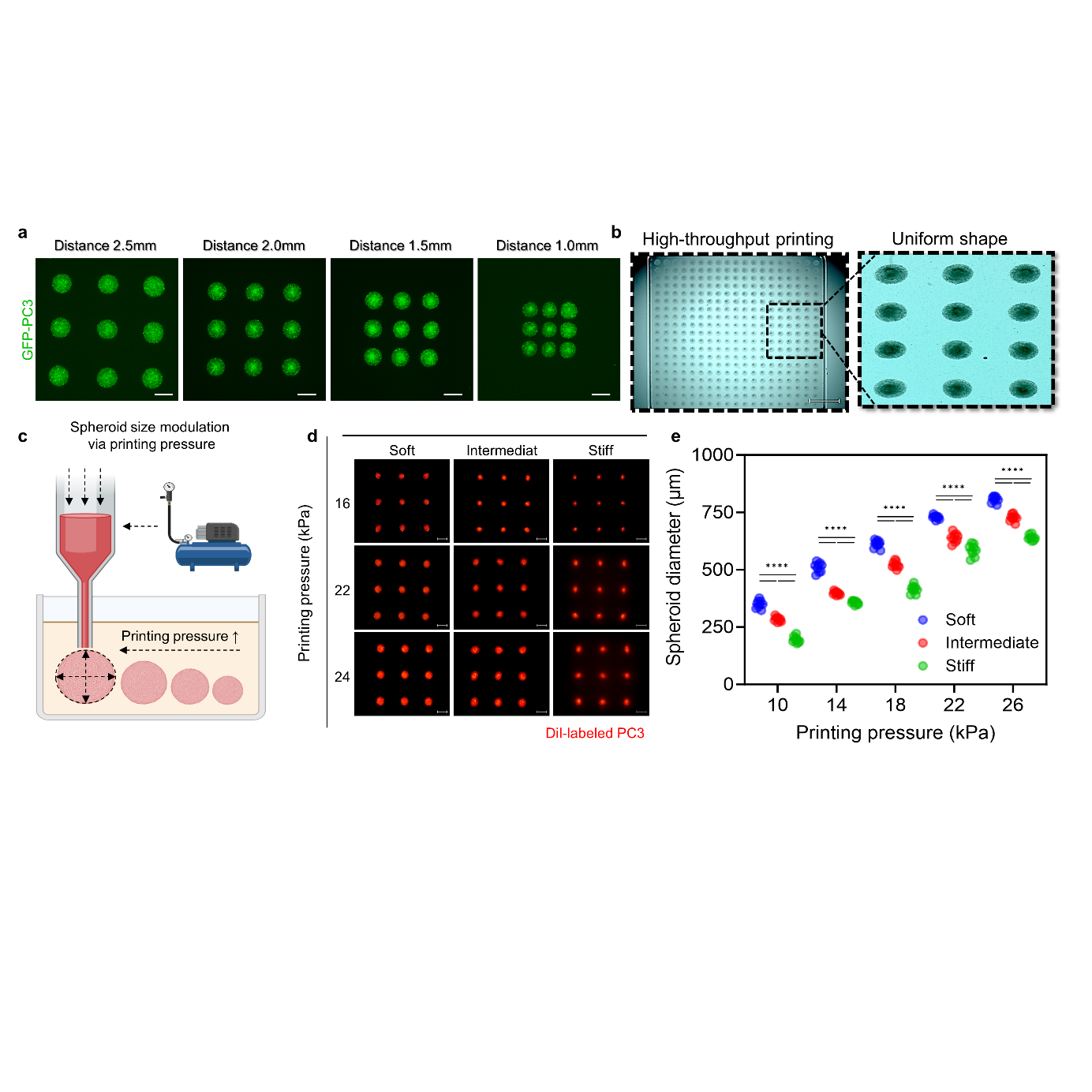


**Supplementary Fig. 4 | Establishment of in–bath bioprinting process.**
(a) Fluorescence microscopy image showing precise positional control based on G–code design. (b) Representative image of high-throughput printing in a 20 × 20 array format. (c) Schematic of in–bath printing process. (d) Fluorescence microscopy images showing spheroid size differences under varying printing pressures for each hybrid bioink. (e) Quantification of spheroid size according to applied printing pressure (n = 9). The error bars represent mean ± SD. Statistical significance was assessed using one-way ANOVA (*p < 0.05, **p < 0.01, ***p < 0.001, ****p < 0.0001).

**
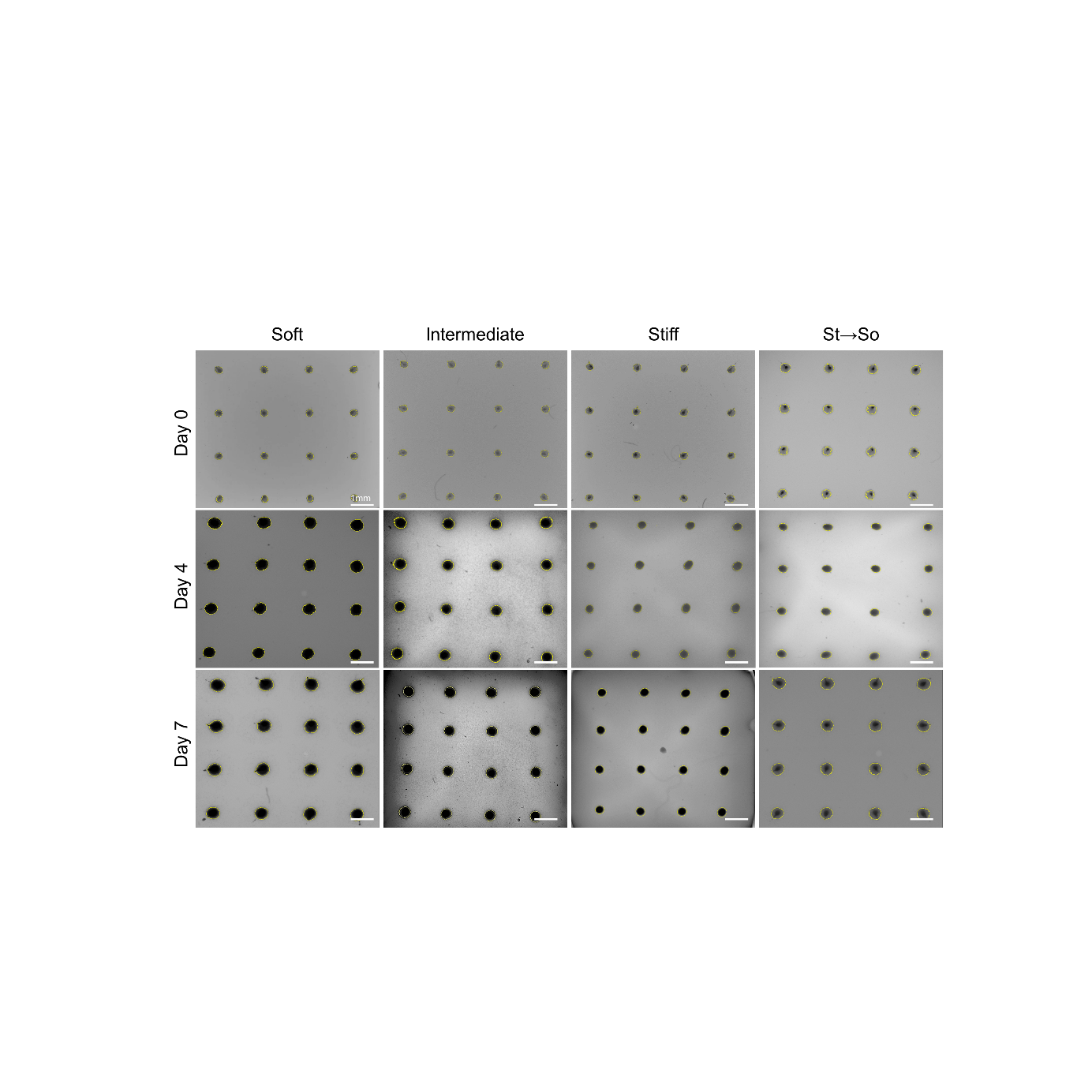
**

**Supplementary Fig. 5 | Comparative analysis of cell expansion across different hybrid bioink groups.** Representative optical microscopy images showing cell expansion in each bioink group. Each group is printed in a 4 × 4 array format and cultured under identical conditions.

**
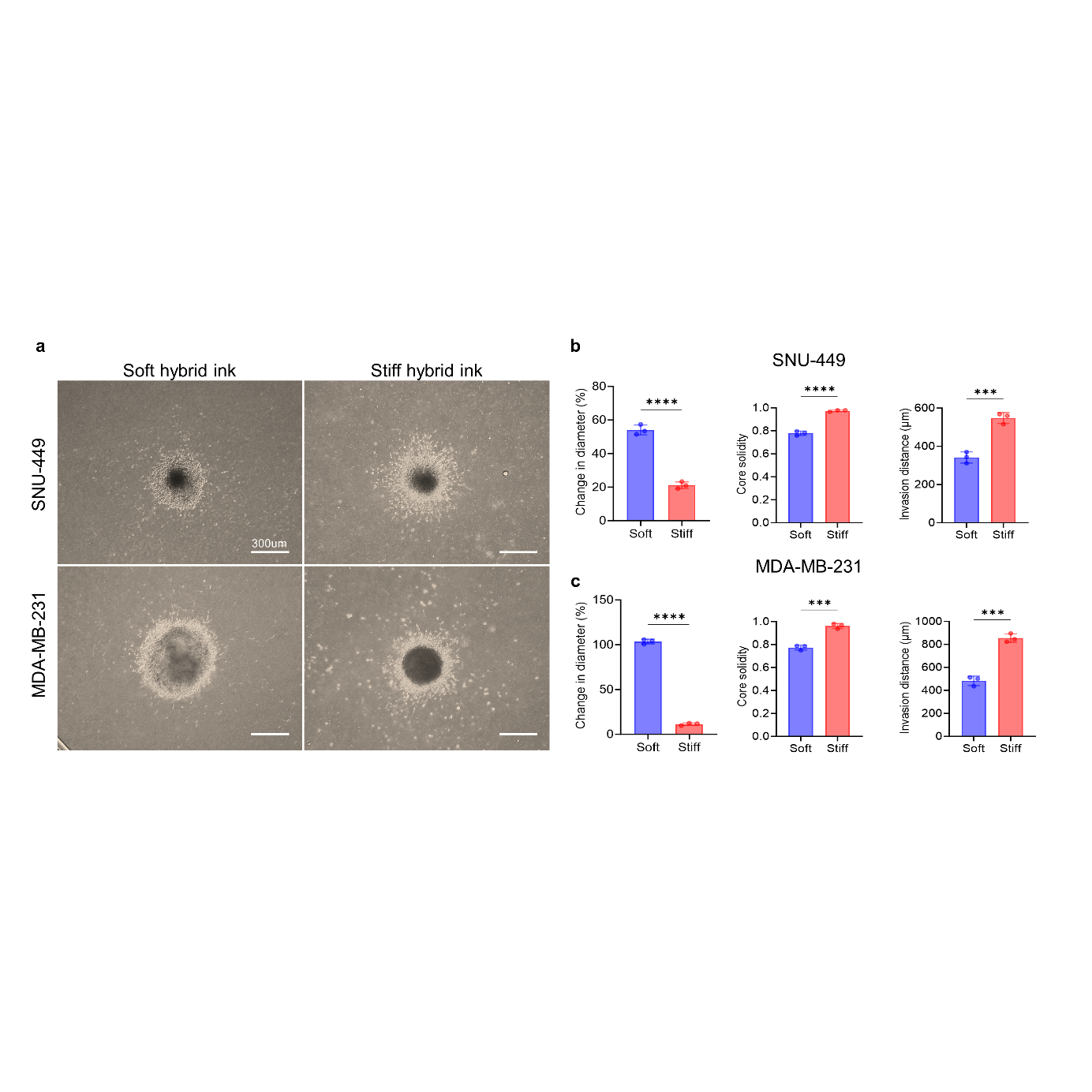
**

**Supplementary Fig. 6 | Comparative analysis of cellular responses across different cancer cell lines within the developed system.** (a) Representative optical microscopy images of SNU–449 and MDA–MB–231 spheroids on day 7. (b) Quantification of spheroid diameter (left), solidity (center), and migration distance (right) for SNU–449 cell line (n = 3). (c) Quantification of spheroid diameter (left), solidity (center), and migration distance (right) for MDA–MB–231 cell line (n = 3). The error bars represent mean ± SD. Statistical significance was assessed using one-way ANOVA (*p < 0.05, **p < 0.01, ***p < 0.001, ****p < 0.0001).


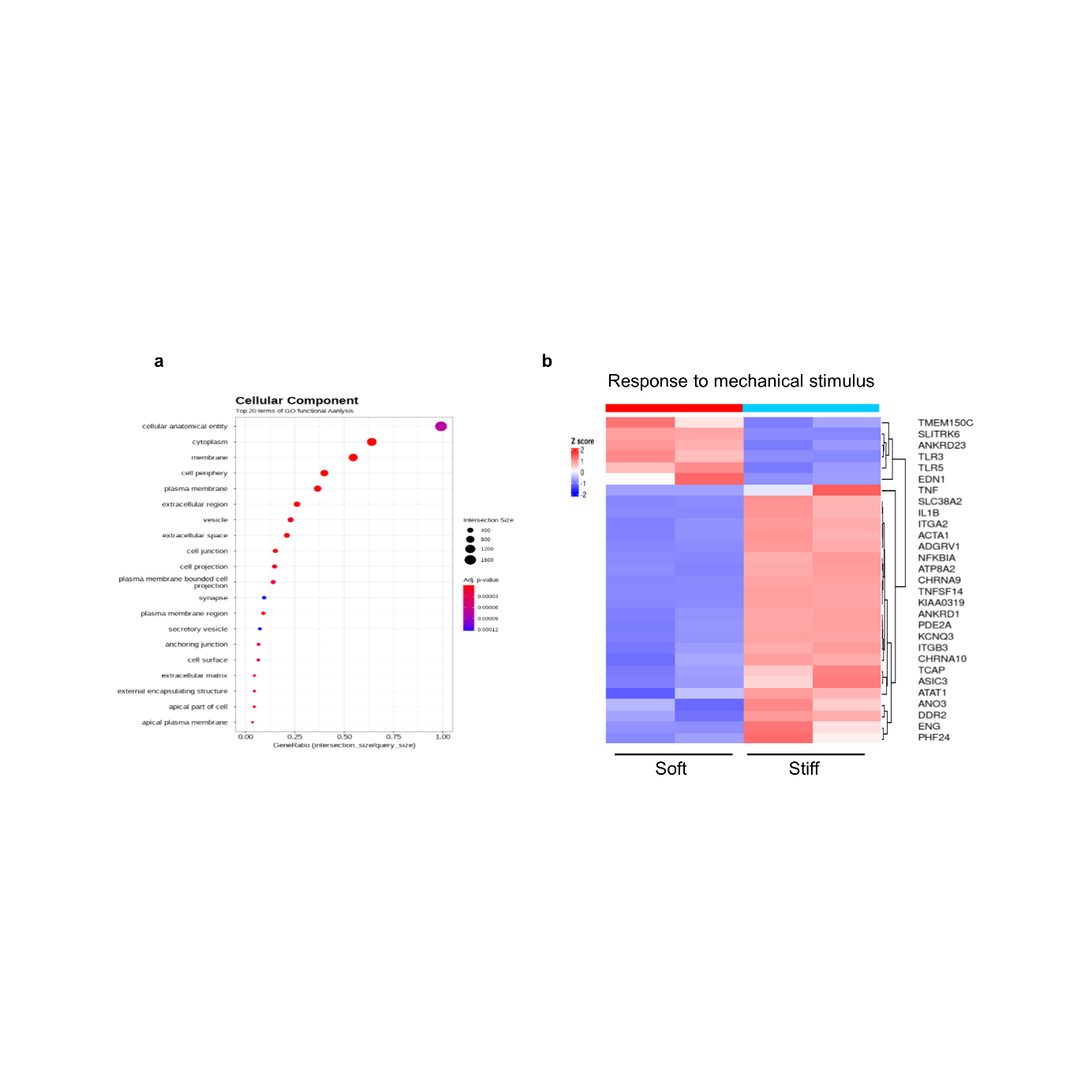


**Supplementary Fig. 7 | Stiffness–induced gene expression changes in the hybrid bioink system.** (a) GO enrichment analysis of genes upregulated in 3D Stiff model, categorized by cellular component (CC) terms. (b) Heatmap analysis of genes associated with “response to mechanical stimulus” category.


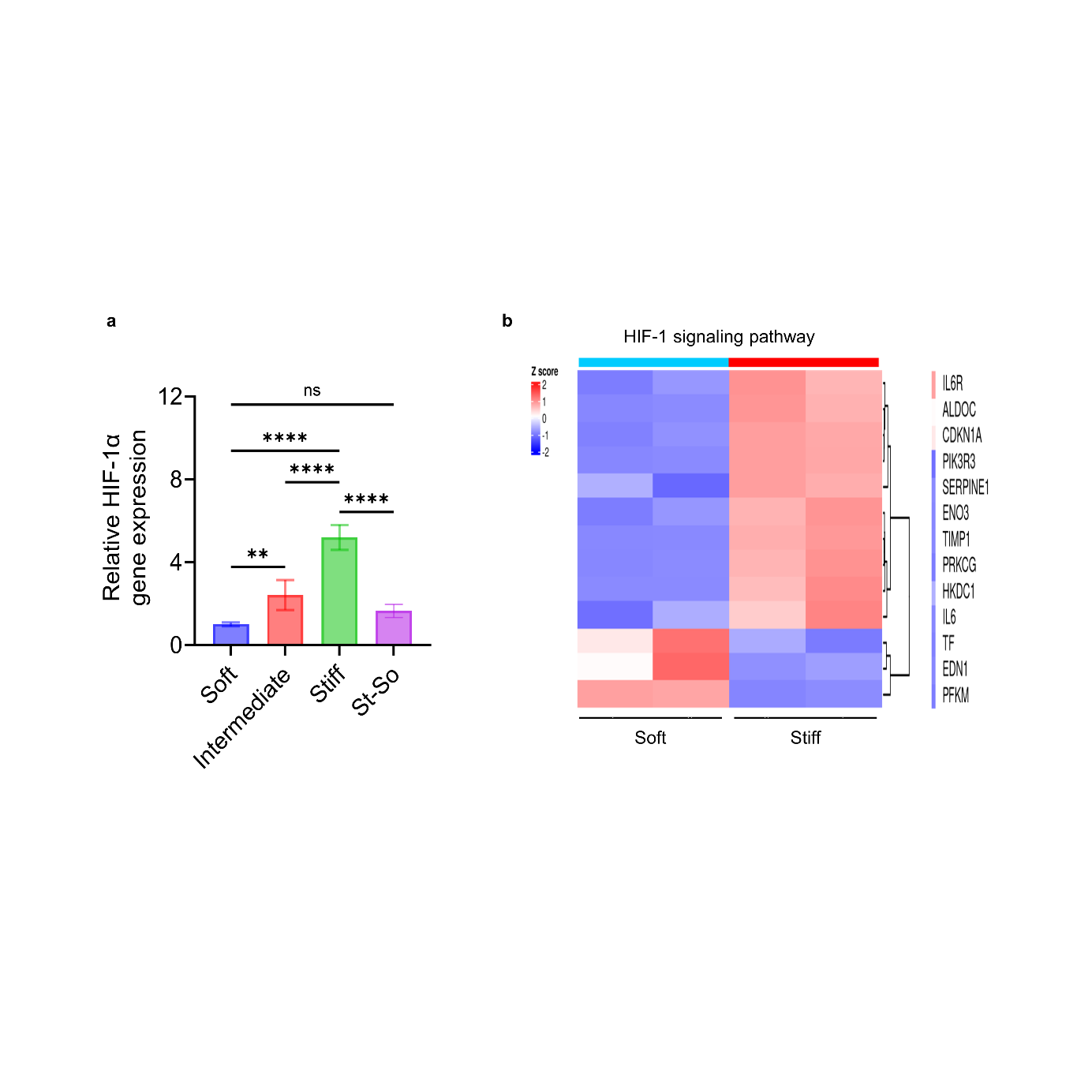


**Supplementary Fig. 8 | Analysis of stiffness–induced hypoxia.** (a) Quantification of relative HIF–1α expression levels across experimental groups (n = 3). (b) Heatmap analysis of genes associated with HIF–1 signaling pathway category. The error bars represent mean ± SD. Statistical significance was assessed using one-way ANOVA (*p < 0.05, **p < 0.01, ***p < 0.001, ****p < 0.0001).


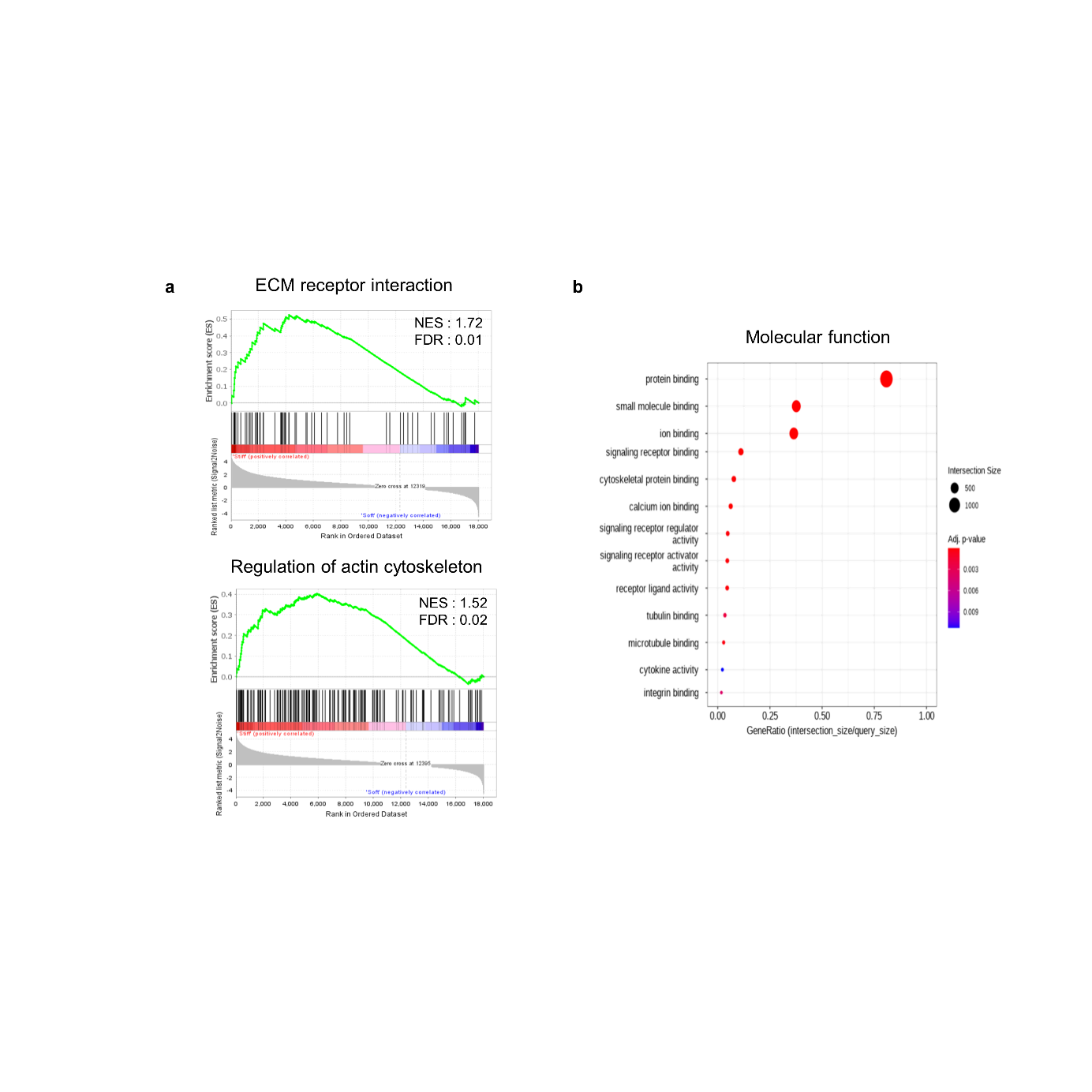


**Supplementary Fig. 9 | Gene expression analysis related to mechanotransduction.** (a) GSEA of ECM–receptor interaction and regulation of actin cytoskeleton pathways. (b) GO enrichment analysis of genes upregulated in 3D Stiff model, categorized by molecular function (MF) terms.


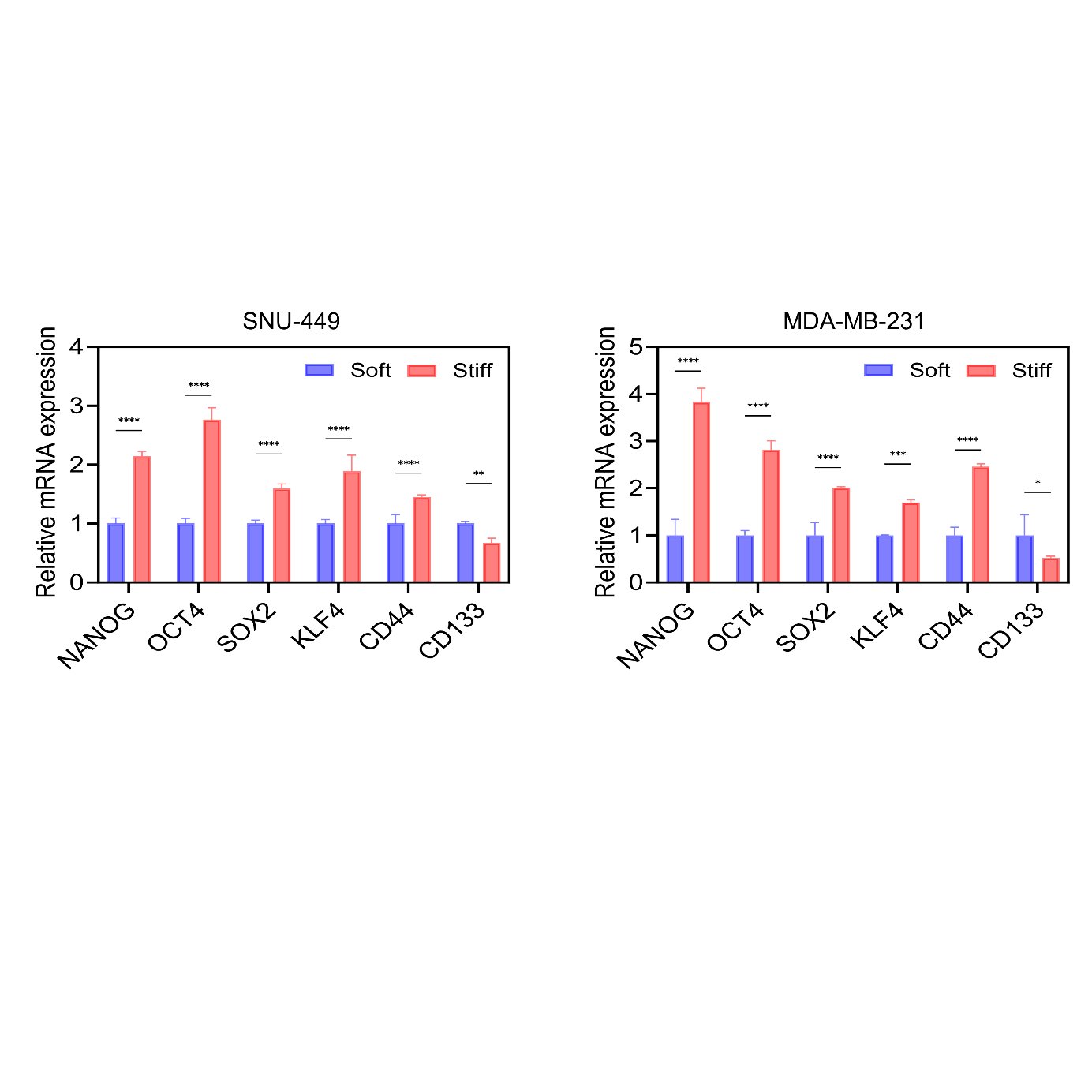


**Supplementary Fig. 10 | Comparative analysis of stiffness–induced changes in stemness markers across different cancer cell lines.** Relative expression of stemness markers in response to stiffness in SNU–449 (left) and MDA–MB–231 (right) cell lines (n = 3). The error bars represent mean ± SD. Statistical significance was assessed using one-way ANOVA (*p < 0.05, **p < 0.01, ***p < 0.001, ****p < 0.0001).


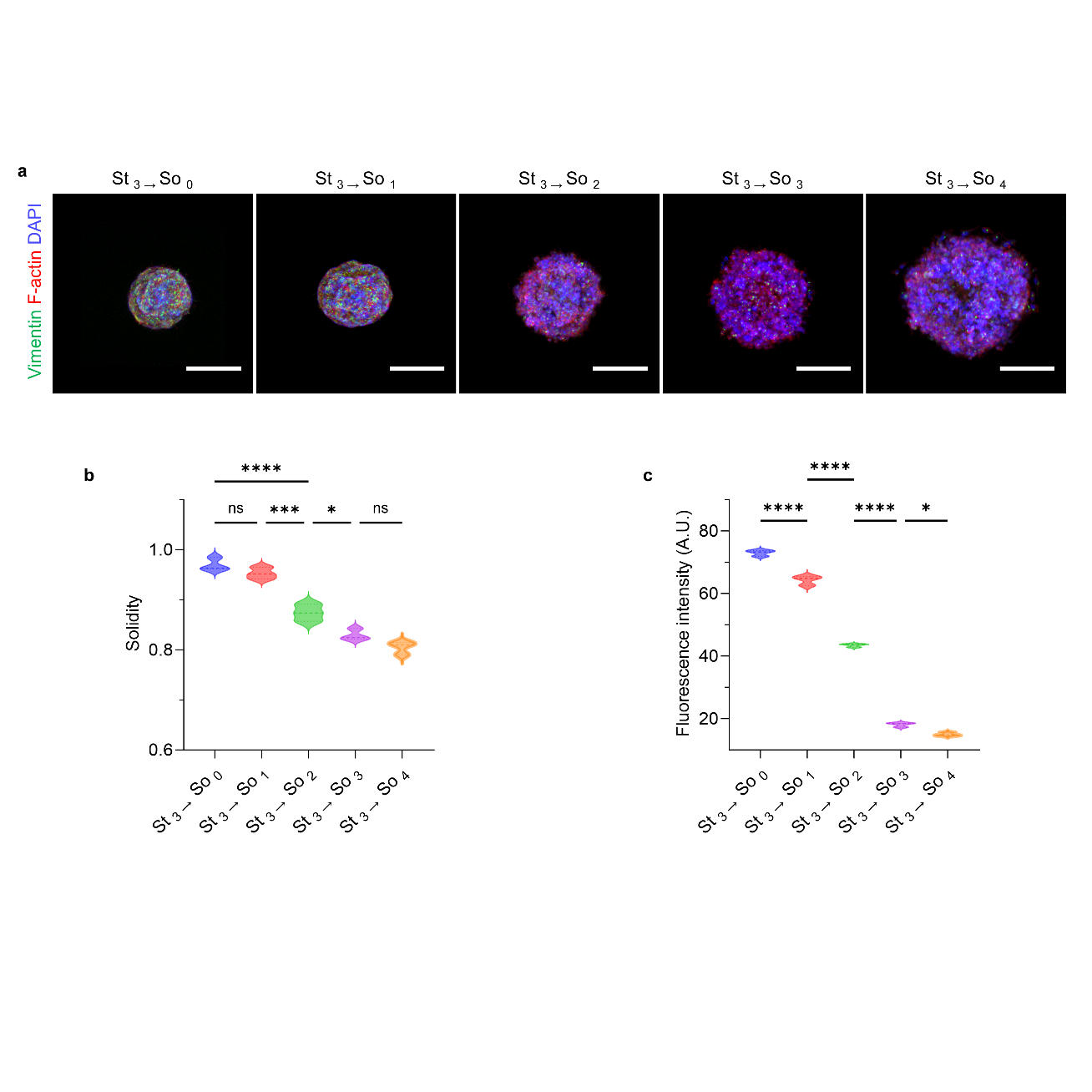


**Supplementary Fig. 11 | Time-dependent structural and EMT phenotypic reversion during the Stiff-to-Soft transition. (a) Representative immunofluorescence images of spheroids at St₃→So₀ to St₃→So₄, stained for Vimentin (green), F-actin (red), and DAPI (blue). Scale bars, 300 µm** (n = 3)**.** **(b) Quantification of spheroid solidity. (c) Quantification of vimentin fluorescence intensity** (n = 3)**.** The error bars represent mean ± SD. Statistical significance was assessed using one-way ANOVA (*p < 0.05, **p < 0.01, ***p < 0.001, ****p < 0.0001).


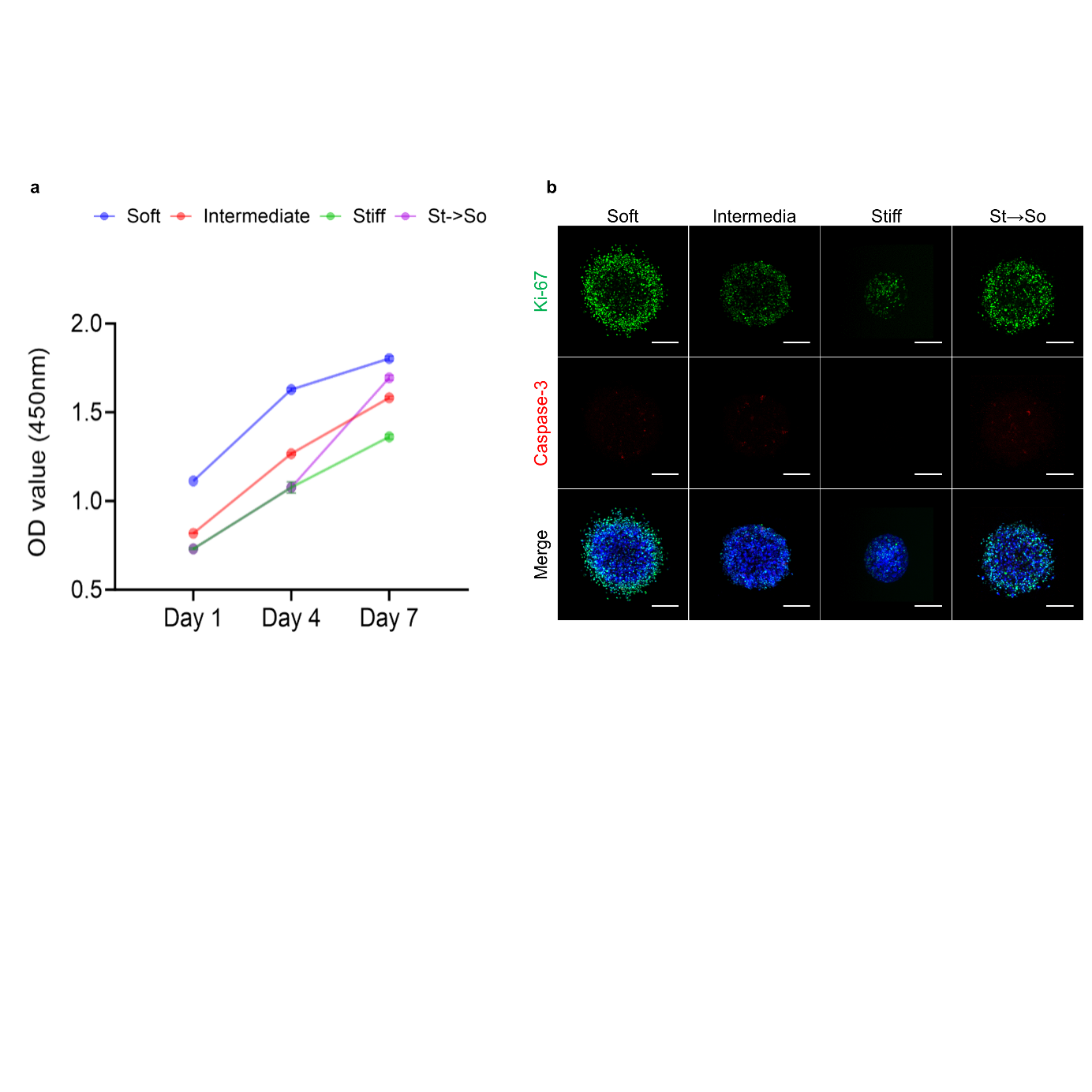


**Supplementary Fig. 12 | Comparative analysis of cell proliferation rates.** Cell proliferation at days 1, 4, and 7 is assessed using the CCK–8 assay across experimental groups (n = 3). (b) Representative confocal images of Ki-67 (green), Caspase-3(red) and DAPI (blue) staining in each group. Scale bars, 200 μm.


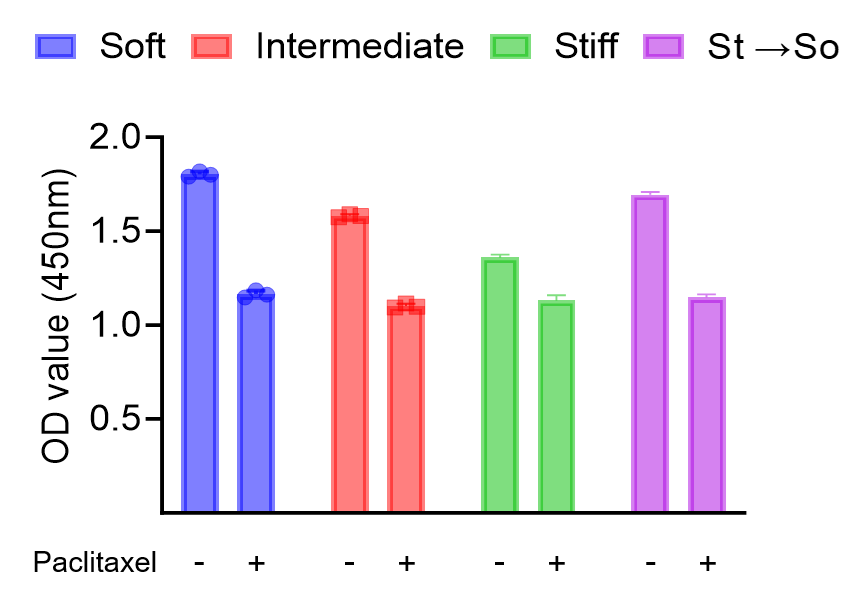


**Supplementary Fig. 13 | Comparative analysis of dynamic proliferation rates under paclitaxel treatment.** Cell proliferation at day 7 following paclitaxel treatment is assessed using the CCK–8 assay across experimental groups (n = 3). The error bars represent mean ± SD. Statistical significance was assessed using one-way ANOVA (*p < 0.05, **p < 0.01, ***p < 0.001, ****p < 0.0001).


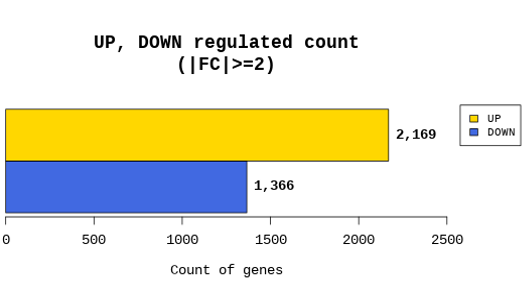


**Supplementary Fig. 14 | Differential gene expression analysis according to stiffness.**
Quantification of number of upregulated and downregulated genes in Stiff group relative to Soft group.


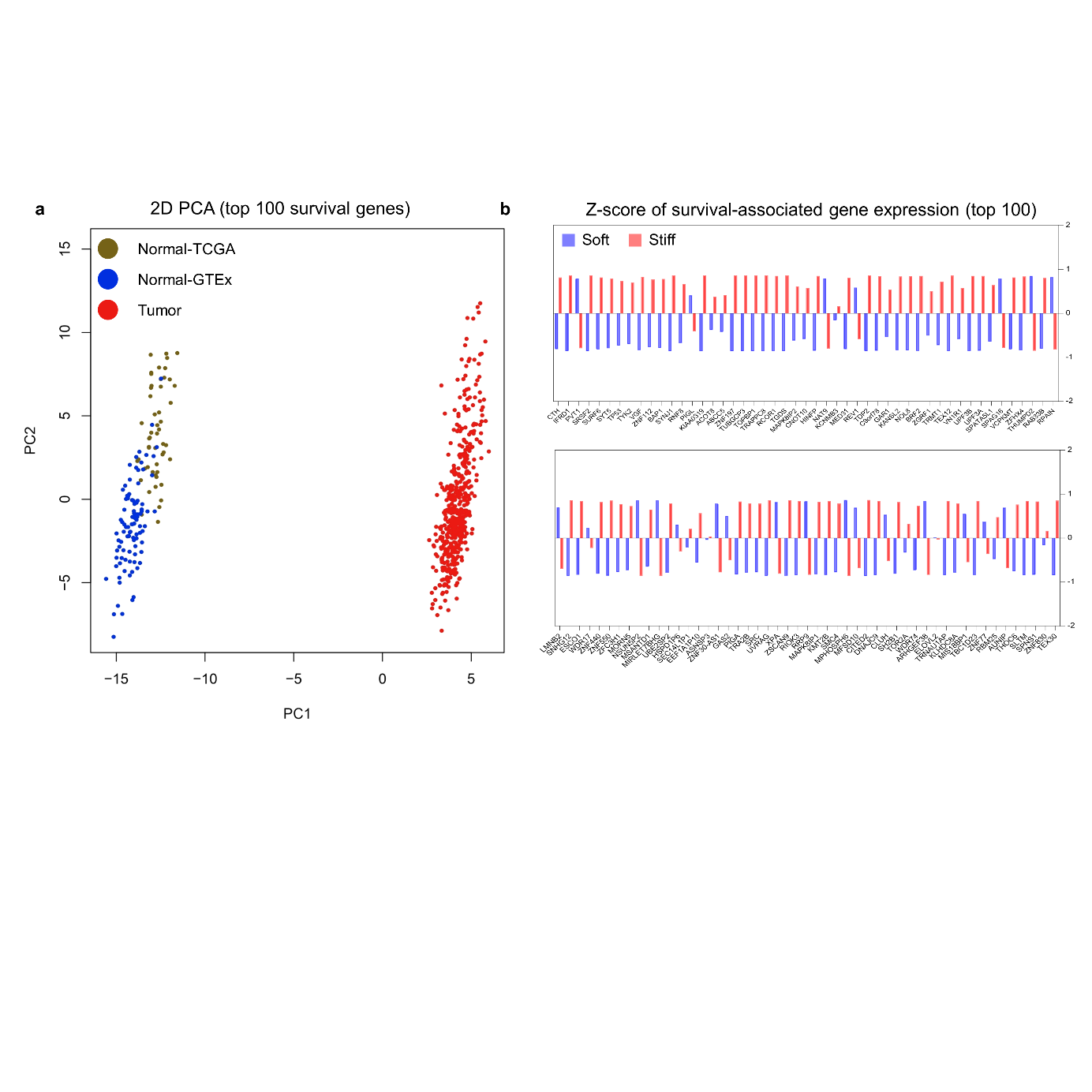


**Supplementary Fig. 15 | Application of a clinical survival-associated gene signature to the stiffness-driven malignant model.** (a) 2D PCA plot of the top 100 survival-associated genes derived from GEPIA2 database, showing separation between tumor (n=492) and normal samples (n=152). (b) Z-score comparison of the same 100 genes in soft and stiff groups, showing 77 genes upregulated under stiff conditions.


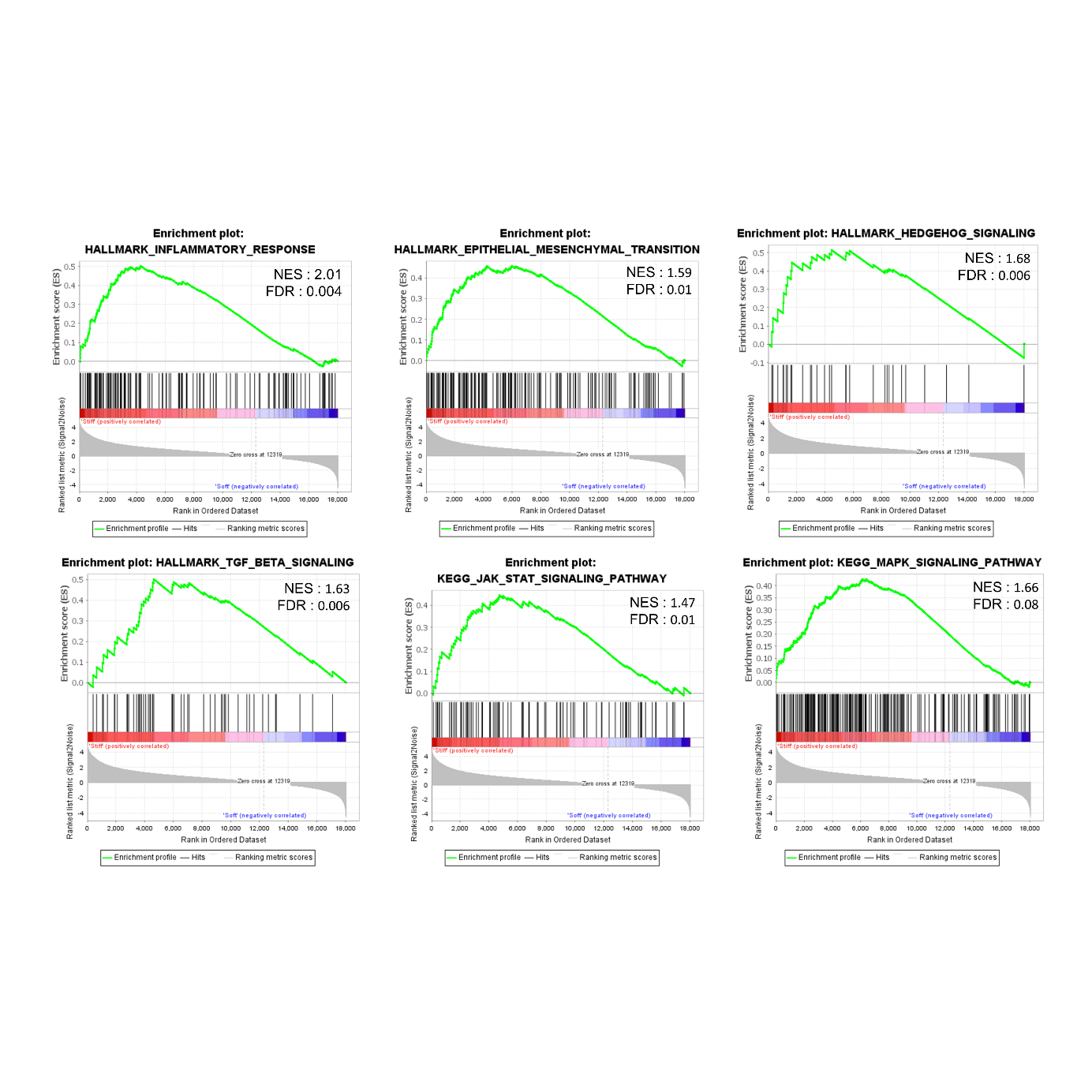


**Supplementary Fig. 16 | GSEA of stiffness–induced transcriptional profiles.** GSEA revealed significant enrichment of multiple pathways in stiff group, including hallmark_inflammatory_response (NES = 2.01, FDR = 0.004), hallmark_epithelial_mesenchymal_transition (NES = 1.59, FDR = 0.01), hallmark_hedgehog_signaling (NES = 1.68, FDR = 0.006), hallmark_tgf_beta_signaling (NES = 1.63, FDR = 0.006), kegg_jak_stat_signaling_pathway (NES = 1.47, FDR = 0.01), and kegg_mapk_signaling_pathway (NES = 1.66, FDR = 0.08).


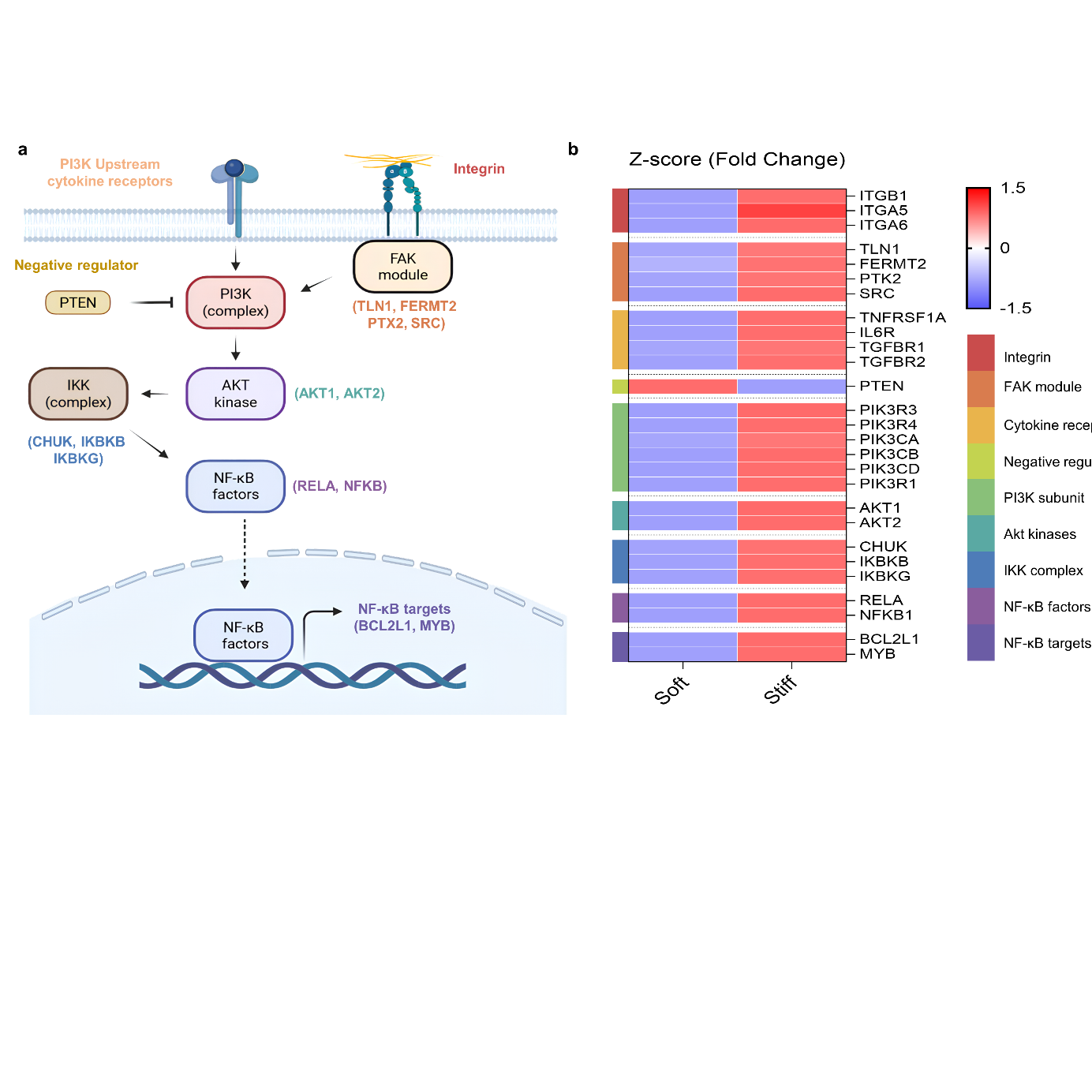


**Supplementary Fig. 17 | Gene expression profiling of the PI3K/NF-κB signaling cascade under stiffness conditions.** (a) Schematic overview of key components in the PI3K/NF-κB pathway. (b) Heatmap showing the expression profiles of the corresponding genes under different stiffness conditions.

**
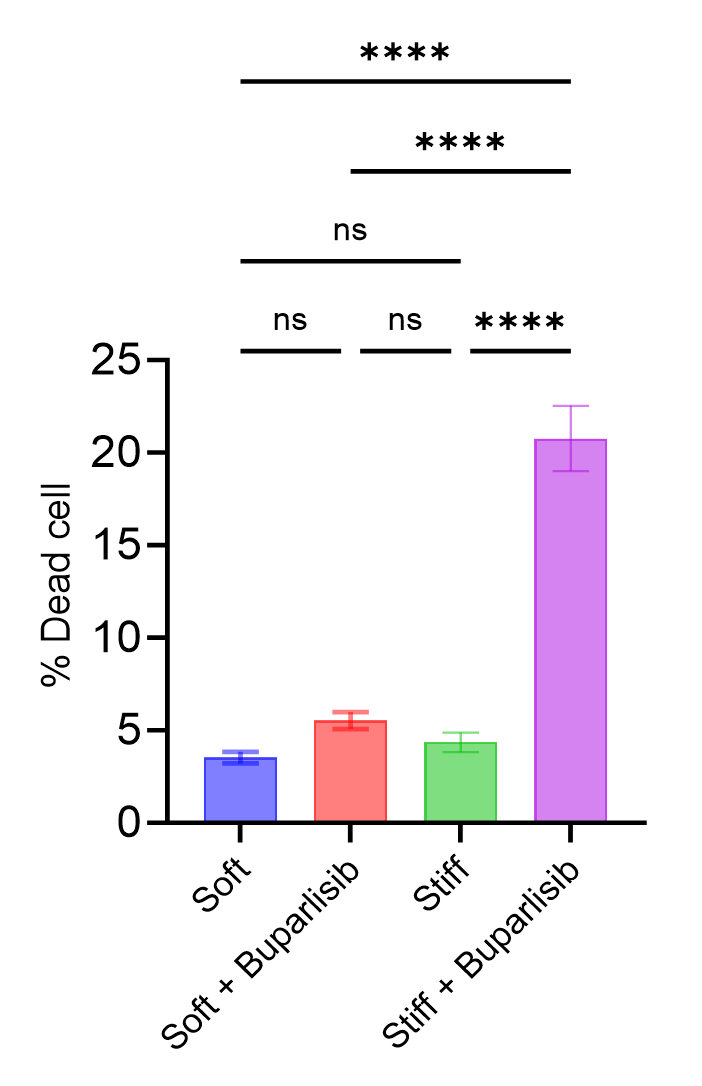
**

**Supplementary Fig. 18 | Assessment of cell viability following buparlisib monotherapy.**
Cell viability is quantified for 2 d after treatment with 25 μM buparlisib across each stiffness condition group, to evaluate the cytotoxic effect of PI3K inhibition (n = 3). The error bars represent mean ± SD. Statistical significance was assessed using one-way ANOVA (*p < 0.05, **p < 0.01, ***p < 0.001, ****p < 0.0001).


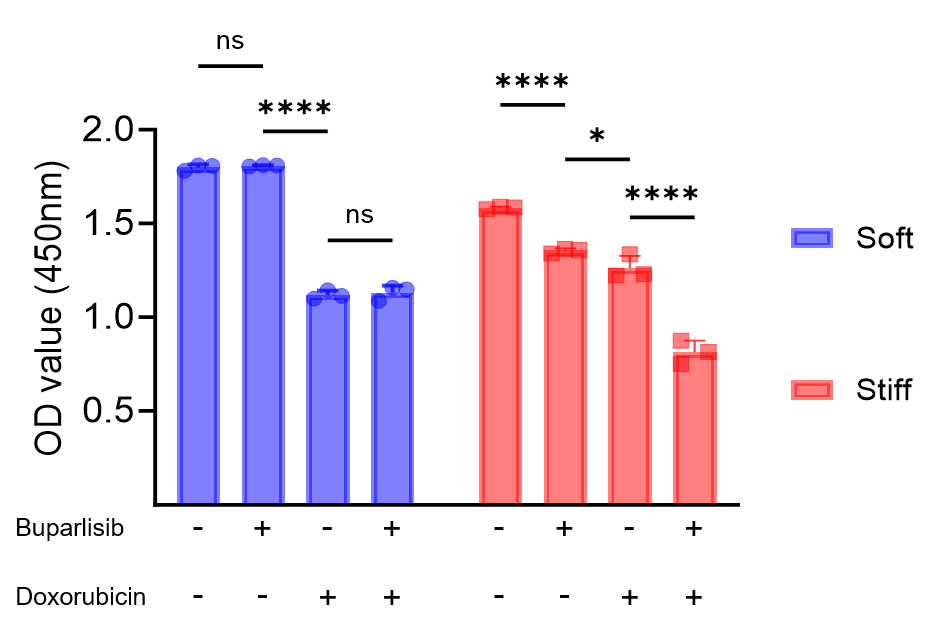


**Supplementary Fig. 19 | Comparative analysis of dynamic proliferation rates under PI3K inhibition and doxorubicin treatment.** Cell proliferation at day 7 under soft and stiff conditions with or without buparlisib and doxorubicin treatment is assessed using the CCK–8 assay (n = 3). The error bars represent mean ± SD. Statistical significance was assessed using one-way ANOVA (*p < 0.05, **p < 0.01, ***p < 0.001, ****p < 0.0001).
